# Vulnerability in research ethics: A call for assessing vulnerability and implementing protections

**DOI:** 10.1073/pnas.2322821121

**Published:** 2024-08-14

**Authors:** Michael G. Findley, Faten Ghosn, Sara J. Lowe

**Affiliations:** ^a^Department of Government, University of Texas at Austin, Austin, TX 78712; ^b^Department of Government, University of Essex, Colchester CO4 3SQ, United Kingdom; ^c^Innovations for Peace and Development, University of Texas at Austin, Austin, TX 78712

**Keywords:** vulnerability, ethics, human subjects

## Abstract

Ethics standards reference the need for special consideration of vulnerable populations, such as pregnant women, incarcerated individuals, and minors. The concept of vulnerability is poorly conceptualized in the medical sciences where it originated, and its application to the social sciences is even more challenging. Social science researchers may unwittingly fail to appreciate preexisting vulnerabilities and indeed may be responsible for inducing new research-related vulnerability. In this paper, we present the first comprehensive coding of country-level vulnerability designations. Specifically, we coded all 355 official documents governing social/behavioral human subjects research for the 107 countries with such regulations and identified 68 distinct vulnerability categories. The data reveal substantial regional variation, overemphasis of categories derived from medical sciences, neglect of critical categories such as displacement, and likely heterogeneity within and across groups. The article provides a conceptual framework that shifts the problem away from static, enumerated categories toward emphasis on research-induced vulnerability. Based on our conceptualization and coding, we present a framework for assessing vulnerability and implementing appropriate protections.

## Significance

This article examines the standard approach of relying on stated categories for addressing vulnerability and presents a practical approach for improving vulnerability assessment and protections. Original data collected from 355 legal/regulatory documents governing social/behavioral research for 107 countries identifies 68 distinct vulnerability categories that vary regionally, calling attention to possible overreliance on international standards. The data also reveal that some categories, such as displacement and trafficking, where research participants are vulnerable by any reasonable definition, are neglected. The article provides a conceptual framework that shifts the problem away from static, enumerated categories toward differentiation of inherent, situational, and research-induced vulnerability. Based on our conceptualization and coding, we present a framework for assessing vulnerability and implementing appropriate protections.

Vulnerability as a research ethics consideration is rarely defined and has relied on naming example categories, such as pregnant women or incarcerated individuals. The concept of vulnerability is poorly conceptualized in the medical and health sciences where it originated (1–4), and its application to the social sciences may be even more challenging. Social science researchers may unwittingly fail to appreciate preexisting vulnerabilities not referenced in medical standards, such as, forcibly displaced persons suffering from posttraumatic stress disorder. Researchers may also be responsible for rendering individuals newly vulnerable or exacerbating existing vulnerability (5). As prospective, field-based social science research continues to grow rapidly, interventions on a broader set of individuals and communities, many of whom may already be vulnerable or rendered vulnerable by the research, need greater attention.

A discussion is currently underway about how to modify the ethics standards outlined in the Belmont Report (Belmont) and the Code of Federal Regulations (CFR), or more broadly others such as Council for International Organizations of Medical Sciences (CIOMS), The Tri-Council Policy Statement 2: Ethical Conduct for Research Involving Humans (TCPS2), and International Council for Harmonisation (ICH), to make them more suitable for social science research (6–9).^*^ There have been calls for greater attention to societies in addition to subjects, researcher-subject power differentials, retraumatization interventions, and therapeutic activities, among others (10). The American Political Science Association and some journals have begun to update their principles, guidelines, and policies in productive ways (11–13). There is important variation by discipline, however. Economics does not have dedicated ethics standards for human subjects research, and while psychology and sociology updated their standards in 2017 and 2018, they still hue very closely to Belmont and CFR.^†^ Specific attention to vulnerability is extremely limited across the social sciences.

At least 146 countries articulate vulnerability categories in legislative and regulatory documents. Most of these countries (107) provide general and social/behavioral (GSB) ethics standards (not simply medical and health). For these 107 countries, we coded the full set of 355 GSB documents and identified 68 precise vulnerability categories (consolidated from an original list of 153). Country-designated categories are important for many reasons, including that they may be more empirically relevant and represent opportunities to learn about local context and confer respect for local standards. However, such regulations might reflect political biases, lack of capacity, or insufficient concern. Relying on labeled categories is primarily useful to the extent that the categories are collectively exhaustive, mutually exclusive, and unconditionally applicable. This is unlikely to be the case as vulnerability is both relational and dynamic (3), especially to the research context. For example, the frequently cited vulnerability category of pregnant women is likely irrelevant for social science surveys on voting. Alternatively, the category of displaced/trafficked persons rarely appears in any standards but likely represents what vulnerability designations are meant to capture. Moreover, research interventions may exacerbate vulnerability for some individuals and not others even in the same named group.

In this paper, we conceptualize vulnerability as being inherent, situational, and research-induced (5), which makes possible appropriate consideration of the relevant research context. Research can and should occur with individuals and communities with preexisting inherent and situational vulnerabilities so long as researchers take appropriate steps. Importantly, emphasis needs to be shifted toward the characteristics of research studies and the potential they have to induce or exacerbate vulnerability.

Interventions with the potential to render individuals and communities newly vulnerable, or exacerbate existing vulnerabilities, appear with regularity in leading journals. Lab and survey experiments have introduced interventions about one true religion supporters wanting to harm subjects (16), chronic- and contextually activated holocaust exposure (17), violence exposure and recall (18), videos of violence in conflict regions (19), and fear primes within autocratic regimes (20). Field experiments have introduced interventions depriving citizens of democratic rights without consent (21), inducing anxiety through racially charged messages to Black voters (22), allocating cash transfers that have sometimes contributed to intimate partner violence among low-education women (23–25), and depriving extremely low-income Kenyans of household water (26). Vulnerability as an ethics consideration is also important for other prospective research, including ethnographies and surveys, where individuals and communities are affected by the research process itself (27, 28). Retrospective, observational research in which data confidentiality is a concern is also relevant, as evidenced by the recent NYU data leak that exposed the identities of thousands of atrocity survivors and sexual abuse victims in the Democratic Republic of the Congo (29). Not all seemingly problematic research contexts represent ethics violations. Retraumatization or regular questioning about current mental illness, for example, have had some positive effects (30, 31). But without a robust approach, it will be difficult to reach definitive conclusions about the ways in which research-induced vulnerability is a concern.

In this paper, we develop a framework for assessing vulnerability and implementing relevant protections. The framework, which we refer to as TAPIR, outlines the implications of researcher decisions about Topics, steps to Appraise vulnerability, strategies for vulnerability Protections, considerations for Implementation fidelity, and commitments to ongoing Reflection. The framework provides a set of considerations for vulnerability in research ethics, which we hope stimulates a productive discussion and greater attention to ethics evaluation (32). We also discuss how a broad set of stakeholders may create institutional incentives and develop positive norms for engaging vulnerability and ethics.

Our first responsibility should be the protection and promotion of all individuals and communities touched in any way by our research, especially those who are currently vulnerable or could be rendered vulnerable by research. As the Declaration of Helsinki clarifies, the interests of science and society should “never take precedence over considerations related to the well-being of the subject” (33). Ideally, scientific and ethical considerations converge with respect to vulnerability and are taken seriously both procedurally as well as in practice (34). Human-centered design, in genuine collaboration with local actors and context, increases the likelihood that scientific and ethical considerations converge, a practice that has not always been the norm (35). This is also key because the content, quality, and validity of the data that researchers obtain may depend critically on ethical engagement with human subjects and their communities (36). Whether we appeal to ethical considerations or research validity, vulnerability as an ethics consideration needs sustained attention in the social sciences.

## Theory and Practice

The logic of scientific inquiry depends on the notion that the generation of knowledge is beneficial to society, either through the accumulation of scientific knowledge and (or) through the application of scientific findings to public policy (37). One way of conceptualizing this commitment is that the scientific community and society have reached an implicit agreement: Society grants the scientific community license to conduct research under certain conditions including being ethical, and the scientific community, for its part, uses the findings from the research for society’s benefit (38). Ethics standards represent approximations of the key parts of the agreement and historically require that researchers minimize risk and maximize benefit (beneficence), obtain informed consent (respect for persons), and distribute the benefits and risks of research equitably (justice). Conceptualizing researcher obligations from a consequentialist perspective can be useful, but may privilege the modal research participant over the most vulnerable. A common interpretation of beneficence is that not all individuals need to benefit, and some individuals may face serious risks, which can be justifiable if there are expected improvements for the broader community (39–41). In contrast, deontological work in bioethics, feminist care ethics, and African Ubuntu philosophies emphasizes that human dignity should be the sine qua non of research (42–48). From this perspective, all are vulnerable (49–51) and should be approached uniformly as such regardless of expected outcomes.

Research ethics gained prominence with the Nuremberg Code, which included 10 principles that emphasized the role of consent (52).^‡^ The Declaration of Helsinki, which was first issued in 1964, formalized much of the guidance in the Nuremberg Code, including the emphasis on informed consent, and has since gone through eight revisions, with the most recent version appearing in 2013 (33). The revelation of the Tuskegee Syphilis Study (55) demonstrated that Nuremberg and Helsinki were insufficient. Nuremberg did not provide any mandate for monitoring or enforcement and Helsinki only added a recommendation for institutional review in 1975. The Belmont Report emerged in the United States as a reaction to the failures of Nuremberg and Helsinki and established the central role of institutional review. According to two of its authors, Belmont was not meant to codify specific steps but rather to provide a general, principled moral framework that would prevent future abuses similar to Tuskegee (56, 57). Belmont advanced three general principles—respect for persons, beneficence, and justice—and some examples of how to address them. It did not offer clear guidance on how to resolve conflicts among the principles (58, 59), emphasized the risks of overrepresentation of vulnerable groups at the expense of underrepresentation resulting in less equitable access to any participation-related research benefits (60), and overemphasized direct subjects at the expense of communities and research teams (61).

Research ethics standards continue to provide little guidance on how to approach vulnerability. Although all approaches mandate special consideration of vulnerable individuals and populations, only three of the 11 most prominent standards even offer an explicit definition of vulnerability (62). The U.S. Department of Health and Human Services codified Belmont principles into the Code of Federal Regulations (63) and some other countries have similarly incorporated international standards into legislative and regulatory documents. Even so, the codification of ethical standards continues to follow a similar pattern—increased emphasis on ethics generally but with little dedicated attention to vulnerability.

We conceptualize vulnerability taking on three key forms: inherent (i.e., corporeality or neediness such as cognitive impairment), situational (i.e., relational or context-specific such as political repression), and induced (i.e., through researcher or practitioner intervention). Inherent vulnerability is common even in favorable research settings, such as among commonly surveyed populations. For example, according to the National Institute of Mental Health for 2021, 22.8% of the adult population experienced any mental illness, with 19.1% of adults experiencing anxiety and 8.3% experiencing major depression. The rates are higher among females, those with multiple races, and those in the 18 to 25 y old category.^§^ From our conversations with survey researchers, many of them contend that vulnerability is not a relevant concern for them and amounts to, ironically, ethics overkill. And yet exposure to social pressure or other emotionally evocative interventions may be similar to the undue influence placed on the at-risk populations about which ethics standards caution (10). This is especially important given that it is nearly impossible to provide any mental health resources to online participants (64).

Situational vulnerability is also prevalent in a broad array of research settings. Field research is increasingly common in the developing world, including areas of political insecurity, displacement, and conflict (65), where situational vulnerabilities are present by any sensible characterization, but local context is poorly understood (66, 67). Poverty, low education, and endemic health challenges appear greatest in precisely these conflict-affected regions (68), thereby layering vulnerabilities in ways that severely compound ethics considerations (3).

Research may induce entirely new vulnerabilities in contexts where there is no preexisting vulnerability, or it may exacerbate inherent or situational vulnerabilities (5). In this sense, researchers “share in the duty to avoid identifiable wrongs” (2, 196–7) wherever they could be introduced, recognizing that “some groups and individuals are particularly vulnerable and may have an increased likelihood of being wronged or of incurring additional harm” (33). As Elisabeth Wood observed: “the researcher has to anticipate and be attentive to the ways in which the research itself might complicate the lives and practices of the people being studied” (69).

Although a researcher’s obligation is not to make subjects and society more vulnerable than they already are (5), it is conceivable that research interventions might reduce vulnerability in some contexts (3). Offering an approved therapeutic program coupled with cash assistance, for example, may provide direct vulnerability-reducing benefits (70). Or, there could exist potential indirect benefits for a subject via a credible future program in which the individual would benefit, or in which the individual’s family or community would benefit. Active efforts to reduce vulnerability are valuable, but we caution that they need to be implemented with local guidance and engagement in order to avoid misguided paternalistic campaigns (35).

## Categories and Characteristics

Ethical guidelines currently rely on the identification of “certain vulnerable populations” and encourage researchers to take steps to protect them. The U.S. Code of Federal Regulations (45 CFR 46) designates “children, prisoners, individuals with impaired decision-making capacity, or economically or educationally disadvantaged persons” as well as “pregnant women and fetuses” (63). The use of labeled categories anchors ethics considerations to a small set of preidentified groups. Named vulnerability categories of concern also rely on predominantly Western standards with little attention to country-specific designations that account for local priorities. Unfortunately, the reliance on categories has resulted in conceptual ambiguity that stereotypes groups, often accompanied by the risk-averse decision to exclude them entirely from research (1, 71, 72). Although reliance on vulnerability categories alone is problematic, it is important to take them seriously given legal/regulatory imperatives (11, Principle 11), respect for local ethics priorities (73, 74), and for the safety of collaborators and participants (75).

We coded vulnerability categories from all legislative and regulatory standards worldwide. Our sampling frame is the 2019 International Compilation of Human Research Standards provided by the U.S. Department of Health and Human Services (HHS), which includes documents from countries that have enacted legislation or regulations. The list includes documents for 131 countries, 92 of which have GSB documents. We identified 15 countries outside of the HHS list that have GSB documents, resulting in 107 countries with a total of 355 documents. We report below on lessons learned from coding and analyzing version 1.0 of this database.^¶^ (See *SI Appendix*, *Full Coding Process* for a discussion of the coding procedures, including inclusion/exclusion criteria. *SI Appendix*, Fig. S1 illustrates the selection of countries and documents in a CONSORT-style diagram.)

### Categories.

**Fig. 1.**
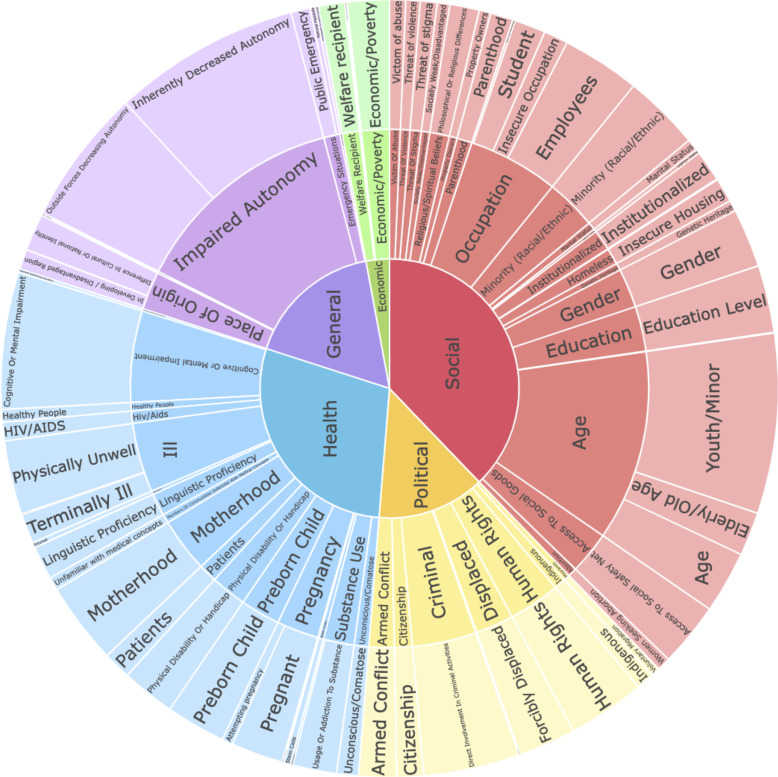
Sunburst of Various Vulnerability Categories. Based on documents from 131 countries of which 107 have general and social/behavioral guidelines. This figure illustrates 68 precise categories (outer ring), 56 general categories (middle ring), and five broad types (inner circle). See R ShinyApp (https://mgfindley.shinyapps.io/vulnerability_10Apr2024/) for a dynamic version of this visualization allowing the user to unpack any broad type to understand its general and precise subtypes.

We coded 153 base vulnerability categories and the number of country documents that contain mention of at least one mention of that particular vulnerability category. *SI Appendix*, Table S1 shows each of the 153 vulnerabilities along with the number of times each is mentioned across countries (no repeats within countries). We aggregated the 153 base categories to 68 precise categories, which grouped similar categories such as “Mothering” and “Mother.” We then grouped those 68 precise categories into 56 general categories, which we then aggregated into five broad types. Fig. 1 illustrates the vulnerability categories at these three levels of aggregation (i.e., precise nested within general nested within broad). The size of each area corresponds to the number of countries that mention the specific category at least once. (The consolidated list of 68 categories appears in *SI Appendix*, Table S2, and the original set of mentions nested into the two highest levels appears in *SI Appendix*, Tables S3 and S4.)

This coding revealed several insights. First, there are far more vulnerability categories than the medical/health literature considered relevant. Our list provides a GSB-based set of categories that should be more suitable for the research questions and settings that are unique to the social sciences.

Second, some vulnerability categories such as those related to humanitarian crises are rarely mentioned. Alas, little has changed since Jacobsen and Landau identified “a general failure to address the ethical problems of researching vulnerable communities” in humanitarian contexts (76). Displaced and trafficked persons are almost never mentioned and yet may be among the most vulnerable by any sensible conceptualization (77, 78). This seems especially critical as there are more displaced persons in the world today (108.4 million) than at any other point in recorded human history and more than double its level only 10 y prior.^#^ The fewest vulnerability mentions are economic with roughly 1% of all mentions in the economic category, and roughly 12% are in the political category.^‖^ The dearth of economic mentions is curious given that economic factors typically characterize vulnerability, at least since Sen’s work on household entitlements (79–83), suggesting a divergence of values between academics and practitioners.

**Fig. 2.**
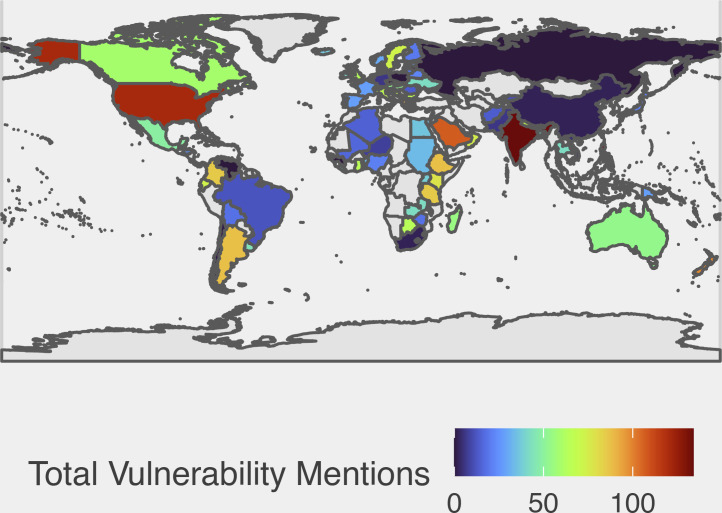
Number of Unique Vulnerability Mentions Globally. (Based on documents from 146 countries of which 107 have general and social/behavioral guidelines. The scale ranges from zero to 131.)

Third, there is substantial regional variation in the vulnerability categories. Fig. 2 illustrates the number of unique vulnerability category mentions globally. (*SI Appendix*, Figs. S2–S7 illustrate the number of unique vulnerability category mentions disaggregated by region. *SI Appendix*, Tables S5–S9 provide the raw overall and sectoral counts by country). India leads the world with the most vulnerability categories (n=131), with 70% of the mentions under the political and social categories. Rwanda has more categories (n=104) than the leading countries in Europe (Greece has 93 categories); despite having 50% of the vulnerable categories fall under the social category, there are zero mentions within the political category. The naming of specific categories might be politically motivated where the exclusion of political rights or displacement, for example, is deliberate. Strikingly, two of the world’s great powers have almost no mentions of GSB vulnerability categories. Russia has zero mentions and China only has two. The fact that Rwanda (an extremely authoritarian country) names many GSB categories but Russia (a great power) names none underscores our point about exclusive reliance on named categories.

Fourth, the sheer number of vulnerability categories means that nearly all research samples will be composed of subjects with differing types and extent of inherent and situational vulnerability even when the research is seemingly innocuous. This raises questions about how to diagnose vulnerability in the presence of such heterogeneity and then how to make decisions about any special protections. Should special protections be implemented based on the modal subject, a large percentage of the subjects, a single subject, or for society? Can special considerations tractably protect individuals and communities with heterogeneously distributed vulnerabilities, and how? Answers to these questions will likely require considerable discussion but need to take place rather than simply assuming homogeneity of a sample or ignoring challenging heterogeneity.

Example categories were never meant to provide the complete scope of vulnerability considerations. In naming categories, some ethics standards also name example characteristics. Those include “increased likelihood of being wronged or of incurring additional harm” (33), “continually be sought as research subjects” (84), or “limited decision-making capacity, or limited access to social goods, such as rights, opportunities, and power” (85). Characteristics are important because they provide guidance about how to identify vulnerability outside of a narrowly named set of categories.

### Characteristics.

The dynamic and relational nature of vulnerability (3) necessitates careful consideration of the relevant characteristics that underpin a particular category, and whether those characteristics are present in a given setting (1, 3, 62, 86). Some vulnerability categories are inherent, such as infancy, and therefore in need of consideration in most or all research situations. However, other categories such as pregnancy would make women vulnerable in the context of a drug trial but not a survey, and more broadly may be sexist (87).

Characteristics of inherent vulnerabilities center on questions of whether individuals and/or groups have the freedom or capacity to consent, are at a suitable development stage, at risk of undue influence or coercion, have experienced harm that could be retriggered, experience ongoing illness that could worsen, have language barriers preventing access to research benefits, or could acquire new inherent vulnerabilities (e.g., new mental illness) during the course of a study. Characteristics of situational vulnerabilities center on questions of whether individuals and/or groups are susceptible to influence in unequal situations, are more submissive and tolerant due to cultural norms, are exploitable due to situations of temporary life-altering circumstances, have been repeatedly exploited historically, are denied the ability to take action to safeguard their own interests, are difficult to reach and cannot access policy/research benefits, or are in environments with multiple overlapping challenges that compound possible harms.

Researchers share in the burden of addressing vulnerability to the extent that research induces new vulnerabilities or exacerbates preexisting vulnerabilities (85, Article 2.8). The identification of research-induced vulnerabilities is difficult because it can be speculative. It entails consideration of whether individuals and/or groups are exposed to untested or early-stage interventions, receive interventions with little field consensus, are subject to always-risky interventions, are frequently/always the target of certain interventions, are promised potentially unrealistically high benefits or low costs, have historically been engaged in unfair or inequitable research, or denied access to possible research benefits because of the overemphasis of risk. Importantly, although many have called out field experiments for ethical violations, vulnerability could manifest in any research setting, such as when subjects disclose sensitive information in the process of an interview or survey, thereby rendering the research situation vulnerable. (*SI Appendix*, Table S10 illustrates a number of characteristics connected to example vulnerability categories, organized by whether they are inherent, situational, or research-induced.)

Whether vulnerability is approached through categories or characteristics, there are both procedural and practical implications. Most emphasis thus far has been on the application of “procedural ethics” whereby researchers seek to satisfy a set of ethical conditions about the proposed research, almost entirely related to commitments to ethics review boards. A better approach would address both procedural and practical ethics over the life cycle of a research project. Taking ethics in practice seriously means embracing calls for greater reflexive openness with respect to the entire research engagement (11, 34, 88).

## Assessment and Protections

We outline a framework that we abbreviate *TAPIR* to capture five key dimensions of addressing vulnerability: Topic, Appraisal, Protection, Implementation, and Reporting. Notably, we intend for this framework to be a starting point that provides structure for broader conceptual and empirical discussions as well as systematic scientific investigation (32). Table 1 summarizes these dimensions.

**Table 1.** Guidance before, during, and after the intervention

Topic: Choose research topics/questions with vulnerability in mind.	
Questions	Choose research questions that will not make subjects or society any worse off than before.
Programs	For existing programs potentially wronging people, study effects of removing components.
Studying up	Ensure that powerful subjects share in the burdens of research.
Abandoning	Set parameters for suspending or abandoning a research topic.
Appraisal: Identify preexisting inherent and situational, and possible research-induced vulnerabilities.	
Assessment	Assess inherent and situational vulnerabilities based on regulatory categories and background research.
Exposure	Conduct exposure assessment for possible research-induced vulnerabilities.
Experiment	Generate rigorous evidence about research-induced vulnerability, if feasible.
Extent	Articulate expectations about the form/extent of induced vulnerabilities to align protections.
Protections: Develop and vet procedural protections for vulnerability.	
Specific protections	Develop protections that may include:
Beneficence	Increase direct benefits and maximize indirect distributive benefits.
Beneficence (cont.)	Tailor sensitive research interventions to reduce direct risk.
Beneficence (cont.)	Provide support services for participants and communities.
Beneficence (cont.)	Develop a compensation procedure for research-induced wrongs.
Beneficence (cont.)	Broaden poststudy access to interventions with proven effects.
Autonomy	Provide accessible and comprehensible consent process outlining unambiguously voluntary participation.
Autonomy	Conduct regular, repeated consent with the same participants.
Autonomy (cont.)	Select a consent approach that leans toward participant autonomy rather than product autonomy.
Autonomy (cont.)	Engage communities as part of the consent process.
Autonomy (cont.)	Implement a fair compensation strategy that is not coercive.
Autonomy (cont.)	Include community voices about protections and dissemination.
Autonomy (cont.)	Improve feedback/complaint mechanisms through better information and greater accessibility.
Justice	Enhance recruitment protocols that prioritize vulnerability in inclusion/exclusion decisions.
Justice (cont.)	Identify and address potential participation barriers to maximize inclusion.
Justice (cont.)	Select research sites that do no “exoticize” particular cultures/peoples.
Justice (cont.)	Obtain community input about the distribution of participants.
Justice (cont.)	Oversample potentially vulnerable when the study has implications for that group.
Justice (cont.)	Plan/monitor/verify the distributive implications of the research, including burden and benefits.
Justice (cont.)	Equitably disseminate research results for improved allocation of future benefits and risks.
Nested incompatibilities	Address incompatibilities between researcher and government/NGO partners.
Vetting	Share ethics plan with the academic, policy, and societal communities.
Advocates	Consider appointing an independent advocate for participants or society.
Documentation	Make ethics plan publicly available ahead of implementation.
Implementation: Implement vulnerability protections in practice.	
Training	Train implementing partners and enumerators on vulnerability assessment and protections.
Monitoring	Monitor implementers/enumerators through checks and reports, and consider an independent monitoring body.
Responsiveness	Credibly commit to adapt, suspend, or terminate a program or evaluation, and report adverse events to IRB.
Privacy	Preserve the privacy of participants throughout the data collection period.
Reflection: Reflect, represent, and report on vulnerability over the project’s life-cycle.	
Verification	Follow up with participants and society to monitor any previously undetected wrongs.
Inclusion	Include the voices of the vulnerable by writing about more than the average participant.
Accountability	Return to subjects and society and report on findings and ethics implementation.
Policy use	Engage with practitioners to encourage responsible policy toward vulnerability.
Confidentiality	Ensure confidentiality through data security and take measures beyond simple deidentification.
Comprehensive reporting	Report fully on the ethics protocol, including any deviations, and discuss lessons for future.

### Topic.

Researchers have latitude in the questions they ask and the populations they study. From a scientific perspective, researchers consider whether a question is answerable. From an ethical perspective, researchers consider whether a question should be answered. Preexisting inherent and situational vulnerabilities could be distributed in many possible ways, and are not themselves the responsibility of researchers. The key consideration is whether proposed research might introduce new vulnerability or exacerbate existing vulnerabilities.

For research questions that may be ethically questionable, researchers might consider alternative approaches. Rather than intervene through the introduction of some treatment, for example, they might instead identify potentially harmful components of existing practices, and study the implications of removing those components. In a study of court fines, for example, researchers intervened by removing court fines/fees (treatment) and compared outcomes to the status quo outcomes from levying fines/fees (control) to understand the effects on otherwise marginalized individuals (89). And rather than always studying conventional populations, which for political science and economics often means those that are marginalized, they might examine decidedly powerful and privileged populations, an approach referred to as “studying up” (90). In a study of illicit finance, for example, the subjects were for-profit financial institutions rather than humans (91).

How we decide to focus on a question and when to abandon one are also important. For example, we may need to draw a distinction between experiments that manipulate the measurement of beliefs, practices, or experiences (e.g., religious beliefs) and experiments that manipulate individuals’ actual beliefs or practices, for while the former may be ethical the latter may not be. So, it is not only “unethical to administer a treatment that turns a subject into an apostate who eventually faces death at the hands of their former religious community, … it [is also] unethical to administer a treatment that turns a subject into a democracy activist who eventually faces imprisonment in a dictatorial regime, even if we believe that spreading democratic norms is good” (92, 42, 57). After selection of a question, there may be times that researchers need to abort the project before moving ahead, which Richard Nielsen modeled (92) after questions were posed to him about the ethics of altering religious beliefs.

### Appraisal.

Once a question is selected, researchers should evaluate the extent to which the research might induce vulnerabilities among anyone affected by the research in any way. Although research could induce vulnerability where it did not previously exist, the research might exacerbate preexisting inherent and situational vulnerabilities for a single individual or a heterogeneously aggregated set of individuals. To assess this possibility, researchers should consider how an intervention (or setting) affects individuals and populations from named categories in international and country-specific standards. Because the categories may not be exhaustive, researchers should also seek out relevant literature and data, and critically assess other possible characteristics of inherent or situational vulnerability that may warrant closer consideration.

As the technology of research advances at such a quick rate, appraisal is complicated in various ways. The rapid rise of recent AI-assisted targeting, for example, raises serious risks of algorithmic exploitation of those most vulnerable (93–95). That is, online recruitment platforms relying on poorly understood algorithms may produce samples with higher-than-average inherent and situational vulnerabilities. Researchers may have an increasingly difficult time assessing preexisting vulnerabilities and monitoring whether their interventions introduce or exacerbate vulnerabilities.

Although past research may provide some insight, there will be uncertainty about the extent of possible research-induced vulnerability, necessitating exposure assessments with a sample of those who could be directly or indirectly affected (96). It is common to pretest instruments or pilot interventions to ensure scientific credibility, but there is no standard practice for gauging possible research-induced vulnerability. In a study that exposed citizens to information about Islamic State of Iraq and Syria (ISIS) collaboration, the authors present a useful example of an exposure assessment wherein they conducted semistructured interviews with Iraqi citizens to anticipate possible retribution or reconciliation better (97). Where there exists mixed evidence about possible vulnerability risks, appropriately designed controlled experiments may be helpful to provide credible evidence for the execution, adaptation, or cessation of such studies (32).

Whether basic exposure assessments or more rigorous experimentation, researchers should document their efforts to diagnose, track, and report on possible vulnerability, including proper accounting of their own possible biases (98). This is broadly consistent with the idea of preregistering one’s “ethical redlines” (99). To the extent that inherent, situational, or induced vulnerability does not appear to be an issue, researchers should document their rationale.

### Protections.

Relevant protections that account for all three forms of vulnerability should then be implemented. Despite its shortcomings, most calls for revision of Belmont emphasize supplementing rather than supplanting the framework so that it, among other things, applies more directly to social research (100) and its provisions extended to societies (10). We thus organize our discussion around the principles of beneficence, respect for persons (autonomy), and justice, but take a deliberately broad perspective that incorporates other insights such as protection of communities and the admonition to engage with greater reflexive openness (e.g., 11, 10, 8, 88).

Specific protections for beneficence include the provision of direct benefits to participants and credible societal benefits distributed to some degree, tailoring sensitive research interventions to reduce direct risk (101), the availability of support services for participants and communities (102), a credible compensation procedure for research-induced wrongs (103), and poststudy access to interventions that have proven effects. Specific protections for autonomy include an accessible and comprehensible consent process outlining unambiguously voluntary participation (104), regular repeated consent with the same participants (105), a consent approach that leans toward participant autonomy rather than product autonomy (7), community involvement in the consent process (106), a fair compensation strategy that is not coercive, community voices about protections and dissemination, and better feedback/complaint mechanisms. Specific protections for justice include enhanced recruitment protocols that prioritize vulnerability considerations in inclusion/exclusion decisions, identification of potential barriers to participation and adaptation to maximize inclusion, site selection that avoids “exoticizing” particular peoples and cultures (106), community input about the distribution of participation, oversampling potentially vulnerable to ensure proper representation when the study has implications for that group, planning/monitoring/verifying the distributive implications of research including its burdens and benefits, and equitable dissemination of research results for the allocation of future benefits and risks.

In addition, we must be aware of “nested incompatibilities” between a researcher’s ethical obligations and that of a partnered research entity, whether that is an non-governmental organization (NGO), intergovernmental organization (IGO), or government. For instance, although a researcher has to request consent and stop an interview if a subject requests, a government official may not have to as long as they do not violate the legal rights of the individual (7, 107). As a result, the researcher and partners need to be clear from the outset who “shoulders moral and legal responsibility for the intervention,” a “sphere of ethics” principle (7, 98) that helps avoid a passing of the buck problem.

Once developed, researchers should share their proposed protections with relevant scholarly and policy communities, as well as with members or representatives of the study populations. Whether through formal feedback events or private communication, a shared discussion about vulnerability protections provides useful guidance and accountability.

Given professional demands, scholars may have no incentive to alter research protocols in light of induced wrongs against subject or society (108). An independent advocate for participants or society might be necessary, especially for those who are unable to advocate for their interests, which may then be unjustly considered (4).

Researchers should document in advance their rationale for conducting research that could increase vulnerability, their approach for assessing existing and possibly induced vulnerabilities, and their proposed protections. This could occur as part of a preregistration plan or in some other public, prospective document. Such documentation need not identify the “truth value” of some diagnosis or prognosis, but rather emphasize transparency of assessment, a logically connected rationale for protections, and a clearly defined accountability process even if self-imposed (109).

To the extent that expected direct research benefits are high, otherwise nonvulnerable subjects should not necessarily be prioritized in such a justification. If researchers take active steps to attempt to reduce vulnerability, they should justify that those steps are not paternalistic or otherwise poorly motivated, which also entails thoughtful justification.

### Implementation.

Protections need to be implemented faithfully and with commitment not only to the integrity of procedural commitments but also to appropriate engagement with vulnerability that arises in practice (34). Principal investigators should train implementers and enumerators on both procedural and practical vulnerability considerations and protections. Even if lead investigators can competently preserve “all the vulnerabilities and proclivities that go along with being human” in their engagement with others (110, 111), it may be difficult to pass this on to implementation and research teams with fidelity.

Given the distance between principal investigators and the implementers or evaluators, monitoring the implementation of vulnerability considerations may be difficult. Even if proper training occurs, monitoring may be difficult to the extent that previously misdiagnosed inherent vulnerability later manifests itself (e.g., mental illness becomes more acute) or situational vulnerability later emerges (e.g., onset of insecurity mid-study). Researchers may need to specify their approach for appraising changing vulnerability, similar to procedures for addressing unexpected research changes (112). A comprehensive vulnerability monitoring plan should thus include some combination of implementer/evaluator reporting, independent researcher checks, and procedures for appraising changes in vulnerability, perhaps overseen by an independent body (113).

The training and monitoring should provide the basis for decisions to adapt, suspend, or terminate a study, which researchers should be prepared to do. There are few professional incentives for a researcher to do this and, by one estimate, precious few studies ever discontinue (114). If research induces vulnerability in serious ways, but researchers do not suspend or cease the study, a logic for adapting the ethical standards needs clear articulation. In general, social science disciplines need to develop better incentives and norms to address such ethics implementation challenges especially when vulnerability is in question.

Throughout a research process, whether or not adaptations are necessary, privacy of research subjects must be paramount. While privacy is an important consideration for all research-affected, the consequences of compromising privacy during research are likely the most detrimental to those who are vulnerable.

### Reflection.

At study completion, there should be dedicated verification that the research did not introduce new vulnerability or took appropriate steps to address it. Given that research-induced vulnerabilities may not manifest for some time, the sort of immediate endline surveys often conducted may not pick up on possible concerns, necessitating longer-term assessment of possible negative effects (for a useful example, see 115). Thus, researchers should verify even if no suspicions were raised earlier and even if a study has global positive effects, as some individuals could nonetheless be wronged in severe ways (103).

Social scientists typically report modal effects, encoded as some form of mean-value statistics, which may depersonalize research effects. The implications for empirical questions are a separate consideration. From an ethics perspective, even though there may be few incentives to highlight vulnerable voices, it may be most important for them. If some individuals and groups stake more on their participation than most, then their voices should emerge even if they are a decided minority of a research sample.

Given that the costs and benefits of research participation may be most pronounced in cases of vulnerability, researchers should return and report on scientific findings that may be the basis for community action or public policy changes. Engagement with the individuals and communities that participated in research is broadly in line with the useful advice to give back to communities that participate in our research, especially when they may not be informed ahead of time (116). If scientific findings might result in negative policy or behavior, decisions to return and disseminate should carefully consider and appropriately justify the rationale, giving special care not to reach these decisions for paternalistic reasons.

Related, in reporting to policymakers and practitioners, researchers should acknowledge that they only have partial control over use and take care to encourage (and monitor) responsible use of data and findings (109) that might be misappropriated for political gains. For programs with demonstrable benefits, researchers may need to encourage inclusion of vulnerable individuals and groups. For programs with pronounced distributive effects, researchers may need to encourage cautious, targeted engagement that promotes benefits and avoids risks.

As with privacy during research, data confidentiality is essential for those who are vulnerable or may have been rendered vulnerable in a research study. Beyond standard deidentification of data, researchers should ensure that aggregating across data characteristics cannot result in inferred identification that could make those participants vulnerable to human or algorithmic exploitation.

In poststudy reports, scholars should comprehensively discuss vulnerability as a matter of course. If increases in vulnerability were considered unlikely, and verified after the fact, then any such discussion would be brief. If benefits were promised, were they delivered as planned? If risks were meant to be avoided, were they? At minimum, researchers should plan ahead, monitor throughout, and then report afterward. Such reporting would explain adherence to (or deviations from) the protections plan during implementation.

## Making Progress: Stakeholders, Incentives, and Norms

Thus far we have discussed vulnerability assessment and protection from the perspective of the researcher. Because few incentives exist for self-monitoring and self-reporting, and because biases may shape approaches with even the best intentions, a broader set of stakeholders needs to engage vulnerability. We expect that progress will only be achieved as stakeholders establish institutional incentives and at the same time develop stronger norms for ethics, including vulnerability. We consider seven other stakeholder groups–ethics boards, associations/editors/reviewers, research/policy communities, implementers, enumerators, funders, and society—and offer initial ideas about possible incentives and norms that they should consider. Of course, the feasibility of instituting such incentives and developing stronger norms varies considerably and will surely require substantial discussion and reflection. Moreover, as incentives and norms develop, they should be examined critically to maximize progress and minimize unnecessary practices. (*SI Appendix*, Table S11 summarizes the stakeholders and key responsibilities.)

University and local ethics boards should shift the focus of vulnerability to the research setting, asking how research might induce vulnerability for anyone, and then address whether the research exacerbates inherent or situational vulnerabilities. As part of this, ethics boards should incorporate the broader set of international and country-specific vulnerability categories into their reviews. They should also develop better mechanisms to engage experts in specific research topics or regions in order to understand the characteristics of vulnerability that may apply beyond named categories, especially to the extent that those countries do not have their own institutional review boards (117). Although more in-depth review would increase demands on both ethics boards and researchers, these substantive considerations would likely be far more beneficial to research than satisfying otherwise thin legal standards (118).

Professional associations set policies for their members, conferences, and journals and vulnerability considerations should begin at that level. Editors and reviewers now give greater attention to preregistration, preanalysis plans, replication, and other scientific transparency concerns, but this shift has not “been accompanied by similar consternation about the injuries too often directly done in intervention-based research” (119). Editors should require advanced disclosure of TAPIR considerations as well as discussion in published articles or *SI Appendix*. Reviewers should also comment more on research-induced vulnerability as a core ethics challenge and include it in their evaluations of manuscripts. Some editors raise concerns about the costs of monitoring ethics and the (lack of) qualifications to make informed assessments (120). Here, professional associations could do more to set priorities, provide resources, and ensure implementation of ethical practice. In the absence of a defined, resourced mandate from associations, journal editors might begin by requiring some/any discussion judged for its inclusion rather than content. They could also consider steps short of a requirement, such as badges for studies that satisfy certain reporting inclusion criteria. As more training and expertise emerge, ethics consideration could increasingly be assessed for content. Here, small incentives could be used to motivate scholars to engage collectively in the longer-term development of stronger norms.

Researcher and policy communities should create forums for discussion of vulnerability in research. Such approaches could include moderated confidential forums for scholars who are (perhaps rightfully) sensitive about being called out as unethical. Communities could do more to proactively highlight and reward positive ethics practices, though accountability processes should also be developed concurrently. Although community discussions will be essential for educating established researchers, graduate students should be exposed to ethics more systematically as part of their education (121). Finally, the research and policy communities may consider the creation of public spaces for posting ethics plans, something similar to the spaces available for posting preregistration and preanalysis plans.

Implementing partners play a key role in much intervention-based research and interface most directly with participants and communities. To the extent that researchers and implementers are not aligned, regardless of which engages vulnerability most directly, they should develop a written understanding or agreement outlining how vulnerability will be assessed and protected over the cycle of the study (7, 8). There has long been a practice that if governments or other implementers plan to carry out a project, then social scientists may evaluate even if ethically questionable so long as some key steps are taken. We add that a vulnerability TAPIR process should be part of those considerations, and that researchers should report comprehensively about the researcher-implementer relationship and ethics approaches. An evaluation of a military policing program in Colombia that had almost uniformly negative impacts, for example, was premised on this rationale (122) though did not address vulnerability. A more transparent discussion would have at least provided guidance for future researcher-implementer relations in such a precarious security setting.

Enumerators are typically trained in survey methodology, but may not be trained in ethics, especially in how to identify research-induced vulnerability, which may be subtle. In some contexts, enumerators are blinded to aspects of a research design, which is helpful in preventing the introduction of bias into the findings. But by not understanding the research context fully, enumerators may have a harder time identifying vulnerabilities, suggesting that there could be some perceived tension in meeting both scientific and ethical obligations. We are not aware of systematic research investigating the possible tension, but at least some evidence suggests that enumerators can be effectively trained in implementing research ethics (123). As AI has taken off in the era of large-language models, researchers and survey firms are increasingly turning to AI to carry out surveys and even interviews. This raises questions about whether AI enumerators inadvertently increase vulnerability, possibly decrease vulnerability, or whether they can even detect it in the first place.

Funders should require recipients to include ethics reflection in proposals, designs, and final reports with due attention to vulnerability assessment and protections. Researchers at Stanford successfully executed an Ethics and Society Review (ESR) process in partnership with an artificial intelligence research grant program, which required robust ethics engagement from the grant application process through the end of the research life cycle (93). Some funders, such as the International Initiative for Impact Evaluation (3ie), require that grant recipients preregister their work as a matter of course, and could add an ESR component to these requirements.

Society should create input and accountability mechanisms that highlight both ethically exemplary research as well as ethically problematic research. For our purposes, such mechanisms would ideally include vulnerability as a central dimension. Society could also do more to create mechanisms for benefiting from social science research. The Cochrane Library, for example, has served as a clearinghouse for medical information, but there is no credible social science analogue to this. Journalists could also publicize with greater consistency questionable research ethics.

## Conclusion and Moving Forward

Consideration of research ethics is on the rise in social science (e.g., 103, 6, 10), which is an unequivocally positive development. And yet scholars rarely include explicit attention to vulnerability in their studies and it rarely appears in broader surveys of what scholars find important (124, 125).^**^ Although recent disciplinary principles and guidelines typically mention vulnerability, they do so with similar ambiguity as standards that originated in medicine and health. That is, they call for special consideration of vulnerable populations but fail to define it or offer concrete approaches for addressing it. The most common thread in recent critiques is the improvement of consent (7, 8, 124). Conventional consent-based approaches assume a lot about participant autonomy (86, 126) and may apply poorly to communal cultures such as Native Americans (127).

Given that vulnerability can be inherent, situational, and induced, its applicability will vary by research area. Improvement will rely on collective efforts to assess vulnerability, implement protections, and then reflect in research reports in order to spur broader discussion about robust approaches. Our practical aspiration is that all studies include even a brief discussion of ethics in their reports (128) with explicit reference to their vulnerability assessment and protections. Several recent studies conducted research with highly vulnerable populations and provide instructive examples of ethics and vulnerability discussions (78, 129). In some specific research areas like conflict and discrimination, researchers have had already advanced some productive ethics dialog that, in part, addresses vulnerability (69, 130–135). The Advancing Research on Conflict Consortium, for example, now provides training and a collaborative community for purposes of improved ethics consideration. The reach of this discussion is increasing but still in its earliest stages, and it is likely that many researchers still embark on fieldwork in conflict regions because they are attractive tourist or voyeuristic destinations (10, 136).

Future research on vulnerability might address a number of issues that we have raised. First, ethics guidelines have not been subject to much systematic testing and should ideally be investigated systematically. This does not mean jettisoning a rich tradition of philosophical and ethical engagement, but it does mean identifying key testable implications that should hold up to some empirical scrutiny. Second, our expanded set of vulnerability categories usefully incorporates globally relevant vulnerability considerations that should be taken seriously. The country-specific categories could be the product of political dynamics, which would be usefully unpacked for a better understanding of how to apply local considerations. Third, with the increase in collaborative research between academics and practitioners, it will be important to develop guidelines on how to determine responsibility as well as whose ethical principles are being followed and why, all before a study commences (7). Fourth, more work needs to be done to help scholars navigate the complex decisions about when to “call it quits” on a research project if ethical issues arise after the research has commenced, something especially consequential for junior scholars. Finally, as AI is increasingly used for targeting and enumeration, the role of AI in inducing or exacerbating vulnerability will require dedicated attention.

We reiterate that a “vulnerability flag” should not trigger a halt to research or necessarily exclude anyone inherently or situationally vulnerable. Exclusion based on inherent or situational vulnerability risks the unfair paternalistic denial of any research benefits. And inclusion may mitigate the “distortionary effects of erasure (observation and sample selection bias), ignorance (omitted variable bias), and misrepresentation (measurement bias),” which are critical to the conduct of credible research (137, 45). It is thus important that we include vulnerable individuals and societies in research, but with much greater reflexive openness (34, 138). Lee Ann Fujii encapsulated our most fundamental commitment to the vulnerable among us: “When conducting research with human beings, we must remind ourselves that to enter another’s world as a researcher is a privilege, not a right. Wrestling with ethical dilemmas is the price we pay for the privileges we enjoy” (132, 722).

## Supplementary Material

Appendix 01 (PDF)

## Data Availability

Replication files data have been deposited in Dataverse (https://doi.org/10.7910/DVN/FC5IPF) (139).

## Footnotes

^*^CIOMS: https://cioms.ch/publications/product/international-ethical-guidelines-for-health-related-research-involving-humans/; TCPS2: https://ethics.gc.ca/eng/policy-politique_tcps2-eptc2_2022.html; ICH of Technical Requirements for Registration of Pharmaceuticals for Human Use: https://www.ema.europa.eu/en/ich-e6-r2-good-clinical-practice-scientific-guideline.

^†^The Jameel Poverty Action Lab has articulated some ethical guidelines for randomized controlled trials (14) and some individual economists have addressed ethics in broader discussions of research standards (15). Psychology’s Ethical Principles were last updated in 2017 and Sociology’s Code of Ethics was last updated in 2018 (see https://www.apa.org/ethics/code and https://www.asanet.org/about/ethics/).

^‡^The 1947 Nuremberg Code may not have been the earliest version of ethics consideration, however. German regulations officially addressed human subject experimentation in 1931 (53) and possibly even as early as 1900 (54).

^§^Accessed 22 December 2023 at https://www.nimh.nih.gov/health/statistics/.

^¶^Replication files available on the journal’s and author’s websites https://doi.org/10.7910/DVN/FC5IPF. Replication data include country-specific vulnerability categories along with links to the relevant legislative/regulatory documentation.

^#^As of 31 December 2022 based on the UNHCR https://www.unhcr.org/en-us/figures-at-a-glance.html. By contrast, the number of refugees in 2012 (42.75 million) was about the same as it was 20 y earlier in 1992 (45.57 million). Accessed on 30 November 2023.

^‖^In the Americas and the Caribbean, 13% of mentions are political and 1% are economic; in the EU and Central Asia, 9% of mentions are political and 1% are economic; in Asia and Pacific, 13% of mentions are political, and 1% are economic; in the Middle East and North Africa, 12% of mentions are political, and 1% are economic; and in Sub-Saharan Africa, 11% of mentions are political, and 1% are economic.

^**^We coded a sample of 121 preanalysis plans from the Evidence in Governance and Politics registry and found almost no mention of vulnerability and little special consideration of vulnerable populations, despite a large share studying populations with at least some vulnerability designation. Of the 121 preanalysis plans we coded, only 10 of them mentioned vulnerability, and only seven of those discussed any protections. Based on the vulnerability categories discussed below, it appears that at minimum 35 of the preanalysis plans have subjects with some inherent or situational vulnerabilities even though they do not mention vulnerability at all. Preanalysis plans are not dedicated ethics documents but represent one of the only prospective documents that most researchers make available. Still, the coding exercise was not random and represents only a small proportion of preanalysis plans and could therefore be idiosyncratic. That said, we are skeptical that a larger coding would reveal substantially different conclusions.

## References

[1] C. Levine , The limitations of “vulnerability’’ as a protection for human research participants. Am. J. Bioeth. **4**, 44–49 (2004). 10.1080/15265160490497083 16192138

[2] S. A. Hurst, Vulnerability in research and health care; Describing the elephant in the room? Bioethics **22**, 191–202 (2008). 18405317 10.1111/j.1467-8519.2008.00631.x

[3] F. Luna, Elucidating the concept of vulnerability: Layers not labels. Int. J. Fem. Approa. Bioet. **2**, 121–139 (2009).

[4] A. K. Martin, N. Tavaglione, S. Hurst, Resolving the conflict: Clarifying “vulnerability’’ in health care ethics. Kennedy Inst. Ethics J. **24**, 51–72 (2014). 24783324 10.1353/ken.2014.0005

[5] M. M. Lange, W. Rogers, S. Dodds, Vulnerability in research ethics: A way forward. Bioethics **27**, 333–340 (2013). 23718774 10.1111/bioe.12032

[6] S. Desposato, Ethics and Experiments: Problems and Solutions for Social Scientists and Policy Professionals (Routledge, 2016).

[7] M. Humphreys, Reflections on the ethics of social experimentation. J. Glob. Dev. **6**, 87–112 (2015).

[8] T. Phillips, Ethics of field experiments. Annu. Rev. Polit. Sci. **24**, 277–300 (2021).

[9] D. Kapiszewski, E. J. Wood, Ethics, epistemology, and openness in research with human participants. Perspect. Polit. **9**, 1–17 (2022).

[10] R. McDermott, P. K. Hatemi, Ethics in field experimentation: A call to establish new standards to protect the public from unwanted manipulation and real harms. Proc. Natl. Acad. Sci. U.S.A. **117**, 30014–30021 (2020). 33229586 10.1073/pnas.2012021117 PMC7720186

[11] APSA, Principles and guidance for human subjects research (2020). https://connect.apsanet.org/hsr/principles-and-guidance/. Accessed 22 July 2024.

[12] S. W. Austin , Notes from the editors. Am. Polit. Sci. Rev. **115**, v–viii (2021).

[13] A. Arjona, W. Pearlman, Note from editors. Perspect. Polit. **21**, 1155–1160 (2023).

[14] L. Feeney, S. Kopper, A. Sautmann, Ethical conduct of randomized evaluations (2022). https://www.povertyactionlab.org/resource/ethical-conduct-randomized-evaluations. Accessed 22 July 2024.

[15] A. Josephson, J. D. Michler, Research Ethics in Applied Economics: A Practical Guide (Taylor & Francis, 2023).

[16] M. Basedau, S. Gobien, L. Hoffmann, Identity threats and ideas of superiority as drivers of religious violence? Evidence from a survey experiment in Dar es Salaam, Tanzania. J. Peace Res. **59**, 395–408 (2021).

[17] D. Canetti , Collective trauma from the lab to the real world: The effects of the holocaust on contemporary Israeli political cognitions. Polit. Psychol. **39**, 3–21 (2018).

[18] F. Bogliacino, G. Grimalda, P. Ortoleva, P. Ring, Exposure to and recall of violence reduce short-term memory and cognitive control. Proc. Natl. Acad. Sci. U.S.A. **114**, 8505–8510 (2017). 28739904 10.1073/pnas.1704651114 PMC5559026

[19] G. Nair, N. Sambanis, Violence exposure and ethnic identification: Evidence from Kashmir. Int. Organ. **73**, 329–363 (2019).

[20] L. E. Young, The psychology of state repression: Fear and dissent decisions in Zimbabwe. Am. Polit. Sci. Rev. **113**, 140–155 (2019).

[21] K. Casey, A. B. Kamara, N. Meriggi, An experiment in candidate selection. Am. Econ. Rev. **111**, 1575–1612 (2021).

[22] D. Nickerson, I. White, The effect of priming racial in-group norms of participation and racial group conflict on black voter turnout: A field experiment (2013). https://polisci.osu.edu/sites/polisci.osu.edu/files/PrimingGroupConflict.3.15.13.pdf. Accessed 22 July 2024.

[23] G. J. Bobonis, M. González-Brenes, R. Castro, Public transfers and domestic violence: The roles of private information and spousal control. Am. Econ. J. Econ. Policy **5**, 179–205 (2013).

[24] M. Hidrobo, L. Fernald, Cash transfers and domestic violence. J. Health Econ. **32**, 304–319 (2013). 23237793 10.1016/j.jhealeco.2012.11.002

[25] V. Baranov, L. Cameron, D. Contreras Suarez, C. Thibout, Theoretical underpinnings and meta-analysis of the effects of cash transfers on intimate partner violence in low-and middle-income countries. J. Dev. Stud. **57**, 1–25 (2021).

[26] A. Coville, S. Galiani, P. Gertler, S. Yoshida, Financing municipal water and sanitation services in Nairobi’s informal settlements. *Rev. Econ. Stat.* 1–48 (2023), 10.1162/rest_a_01379/117913/Financing-Municipal-Water-and-Sanitation-Services.

[27] J. Krause, The ethics of ethnographic methods in conflict zones. J. Peace Res. **58**, 329–341 (2021). 34040265 10.1177/0022343320971021 PMC8120631

[28] D. R. Stroup, J. P. Goode, On The Outside Looking In: Ethnography and Authoritarianism (Perspectives on Politics, 2023), pp. 1–16.

[29] R. Flummerfelt, N. Turse, “Online Atrocity Database Exposed Thousands of Vulnerable People in Congo”. The Intercept. https://theintercept.com/2023/11/17/congo-hrw-nyusecurity-data/. Accessed 17 November 2023.

[30] A. E. Jaffe, D. DiLillo, L. Hoffman, M. Haikalis, R. E. Dykstra, Does it hurt to ask? A meta-analysis of participant reactions to trauma research. Clin. Psychol. Rev. **40**, 40–56 (2015). 26051308 10.1016/j.cpr.2015.05.004

[31] C. W. Mathias , What’s the harm in asking about suicidal ideation? *Suicide Life Threat Behav.* **42**, 341–351 (2012). 10.1111/j.1943-278X.2012.0095.x PMC3597074 22548324

[32] R. Littman, R. Wolfe, G. Blair, S. Ryan, Evidence required for ethical social science. Science **379**, 247–247 (2023). 36656958 10.1126/science.adf8329

[33] WMA Declaration of Helsinki - Ethical Principles for Medical Research Involving Human Subjects. https://www.wma.net/policies-post/wma-declaration-of-helsinki-ethical-principles-for-medical-research-involving-human-subjects/. Accessed 22 July 2024.

[34] M. Guillemin, L. Gillam, Ethics, reflexivity, and “ethically important moments’’ in research. Qual. Inq. **10**, 261–280 (2004).

[35] J. M. Davis, Manipulating Africa? Aerspectives on the experimental method in the study of African politics. Afr. Aff. **119**, 452–467 (2020).

[36] S. E. Parkinson, (Dis)courtesy bias: “methodological cognates,’’ data validity, and ethics in violence-adjacent research. Comp. Pol. Stud. **55**, 1–31 (2021).

[37] D. E. Stokes, Pasteur’s Quadrant: Basic Science and Technological Innovation (Brookings Institution Press, 1997).

[38] C. H. Coleman, Vulnerability as a regulatory category in human research. J. Law Med. Ethics **37**, 12–18 (2009). 19245598 10.1111/j.1748-720X.2009.00346.x

[39] G. H. De Andrade, M. Bruhn, D. McKenzie, A helping hand or the long arm of the law? Experimental evidence on what governments can do to formalize firms. World Bank Econ. Rev. **30**, 24–54 (2016).

[40] R. Glennerster, S. Powers, Balancing risk and benefit: Ethical tradeoffs in running randomized evaluations. *Oxford Handb. Prof. Econ. Ethics* 366–401 (2016).

[41] A. Brockmeyer, S. Smith, M. Hernandez, S. Kettle, Casting a wider tax net: Experimental evidence from costa rica. Am. Econ. J. Econ. Policy **11**, 55–87 (2019).

[42] E. Lévinas, Totalité et infini: Essai sur l’extériorité (Kluwer Academic, 1961).

[43] R. E. Goodin, Protecting the Vulnerable: A Re-analysis of Our Social Responsibilities (University of Chicago Press, 1986).

[44] D. Callahan, The vulnerability of the human condition. Bioeth. Biolaw **2**, 115–122 (2000).

[45] M. H. Kottow, Vulnerability: What kind of principle is it? Med. Health Care Philos. **7**, 281–287 (2004). 15679020 10.1007/s11019-004-6857-6

[46] P. J. Nickel, Vulnerable populations in research: The case of the seriously ill. Theoret. Med. Bioeth. **27**, 245–264 (2006). 16763881 10.1007/s11017-006-9000-2

[47] M. Letseka, Educating for ubuntu/botho: Lessons from basotho indigenous education. Open J. Philos. **3**, 337 (2013).

[48] D. Engster, Care ethics, dependency, and vulnerability. Ethics Soc. Welf. **13**, 100–114 (2019).

[49] M. A. Fineman, The vulnerable subject and the responsive state. Emory Law J. **60**, 251–275 (2010).

[50] B. S. Turner, Vulnerability and Human Rights (Penn State University Press, 2015).

[51] J. Butler, Frames of War: When Is Life Grievable? (Verso Books, 2016).

[52] J. Katz, The nuremberg code and the nuremberg trial: A reappraisal. Jama **276**, 1662–1666 (1996). 8922453

[53] H. M. Sass, Reichsrundschrift 1931: Pre-nuremberg German regulations concerning new therapy and human experimentation. J. Med. Philos. **8**, 99–112 (1983). 6350522 10.1093/jmp/8.2.99

[54] J. Vollmann, R. Winau, The prussian regulation of 1900: Early ethical standards for human experimentation in Germany. IRB Ethics Hum. Res. **18**, 9–11 (1996). 11654878

[55] E. Y. Adashi, L. B. Walters, J. A. Menikoff, The belmont report at 40: Reckoning with time. Am. J. Public Health **108**, 1345–1348 (2018). 30138058 10.2105/AJPH.2018.304580 PMC6137767

[56] T. L. Beauchamp, *The Origins and Evolution of the Belmont Report* (Georgetown University Press, 2005), pp. 12–25.

[57] A. R. Jonsen, On the Origins and Future of the Belmont Report (Georgetown University Press, 2005), pp. 3–11.

[58] R. M. Veatch, Balancing Ranking, or Simultaneity: Resolving Conflicts among the Belmont Principles (Georgetown University Press, 2005), pp. 184–204.

[59] H. S. Richardson, Balancing Specifying, and Interpreting Bioethical Principles (Georgetown University Press, 2005), pp. 205–227.

[60] S. Sherwin, *Belmont Revisited through a Feminist Lens* (Georgetown University Press, 2005), pp. 148–164.

[61] P. Friesen, L. Kearns, B. Redman, A. L. Caplan, Rethinking the belmont report? Am. J. Bioeth. **17**, 15–21 (2017). 10.1080/15265161.2017.1329482 28661753

[62] D. Bracken-Roche, E. Bell, M. E. Macdonald, E. Racine, The concept of “vulnerability’’ in research ethics: An in-depth analysis of policies and guidelines. Health Res. Policy Syst. **15**, 1–18 (2017). 28173859 10.1186/s12961-016-0164-6 PMC5297186

[63] HHS, 45 cfr 46, Technical report, Department of Health and Human Services (2019). https://www.hhs.gov/ohrp/regulations-and-policy/regulations/45-cfr-46/index.html. Accessed 22 July 2024.

[64] J. Chandler, D. Shapiro, Conducting clinical research using crowdsourced convenience samples. Annu. Rev. Clin. Psychol. **12**, 53–81 (2016). 26772208 10.1146/annurev-clinpsy-021815-093623

[65] J. Driscoll, “Prison states and games of chicken” in *Ethics and Experiments: Problems and Solutions for Social Scientists and Policy Professionals*, S. Desposato Ed. (Routledge, 2016), pp. 81–96.

[66] S. Desposato, “Conclusion and recommendations” in *Ethics and Experiments: Problems and Solutions for Social Scientists and Policy Professionals*, S. Desposato Ed. (Routledge, 2016), pp. 267–289.

[67] C. Corduneanu-Huci, M. T. Dorsch, P. Maarek, What, where, who, and why? An empirical investigation of positionality in political science field experiments. PS Polit. Sci. Polit. **55**, 741–748 (2022).

[68] M. Lake, R. S. Pierotti, A. Alik-Lagrange, Resilience, vulnerability, and social isolation: Barriers to poverty reduction in war. Int. Stud. Quart. **67**, sqad075 (2023).

[69] E. J. Wood, The ethical challenges of field research in conflict zones. Qualit. Sociol. **29**, 373–386 (2006).

[70] C. Blattman, J. C. Jamison, M. Sheridan, Reducing crime and violence: Experimental evidence from cognitive behavioral therapy in Liberia. Am. Econ. Rev. **107**, 1165–1206 (2017). 29553237 10.1257/aer.20150503

[71] M. Lake, S. Majic, R. Maxwell, Summary: Research on Vulnerable and Marginalized Populations (Perspectives on Politics, 2022).

[72] A. M. Jacobs , The qualitative transparency deliberations: Insights and implications. Perspect. Polit. **19**, 171–208 (2021).

[73] J. L. Merolla, R. Madrid, “The value and challenges of using local ethical review in comparative politics experiments” in *Ethics and Experiments: Problems and Solutions for Social Scientists and Policy Professionals*, S. Desposato Ed. (Routledge, 2016), pp. 99–112.

[74] S. Cunow, S. Desposato, “Local review: confronting the brazilian black box” in *Ethics and Experiments: Problems and Solutions for Social Scientists and Policy Professionals*, S. Desposato Ed. (Routledge, 2016), pp. 128–138.

[75] X. Lü, “Ethical challenges in comparative politics experiments in china” in *Ethics and Experiments: Problems and Solutions for Social Scientists and Policy Professionals*, S. Desposato Ed. (Routledge, 2016).

[76] K. Jacobsen, L. B. Landau, The dual imperative in refugee research: Some methodological and ethical considerations in social science research on forced migration. Disasters **27**, 185–206 (2003). 14524045 10.1111/1467-7717.00228

[77] D. Palmer, Ethical issues and their practical application in researching mental health and social care needs with forced migrants. Res. Ethics **4**, 20–25 (2008).

[78] F. Ghosn , The journey home: Violence, anchoring, and refugee decisions to return. Am. Polit. Sci. Rev. **115**, 982–998 (2021).

[79] A. Sen, Inequality Reexamined (Harvard University Press, 1995).

[80] J. C. Ribot, The causal structure of vulnerability: Its application to climate impact analysis. GeoJournal **35**, 119–122 (1995).

[81] A. Sen, Development as Freedom (Anchor, 1999).

[82] M. Baro, T. F. Deubel, Persistent hunger: Perspectives on vulnerability, famine, and food security in sub-saharan Africa. Annu. Rev. Anthropol. **35**, 521–538 (2006).

[83] S. Biswas, S. Nautiyal, A review of socio-economic vulnerability: The emergence of its theoretical concepts, models and methodologies. Nat. Hazards Res. **3**, 563–571 (2023).

[84] HHS, The Belmont Report: Ethical Principles and Guidelines for the Protection of Human Subjects of Research (1979). https://www.hhs.gov/ohrp/regulations-and-policy/belmont-report/index.html. Accessed 22 July 2024. 25951677

[85] Canadian Institutes of Health Research, Natural Sciences and Engineering Research Council of Canada, and Social Sciences and Humanities Research Council of Canada, TriCouncil Policy Statement: Ethical Conduct for Research Involving Humans (2022). https://ethics.gc.ca/eng/documents/tcps2-2022-en.pdf. Accessed 22 July 2024.

[86] K. Kipnis, Ethical and Policy Issues in Research Involving Human Participants (National Bioethics Advisory Commission, Bethesda, MD, 2001), vol. II, pp. 1–13.

[87] M. C. Ruof, Vulnerability, vulnerable populations, and policy. Kenn. Inst. Ethics J. **14**, 411–425 (2004). 10.1353/ken.2004.0044 15812988

[88] L. M. MacLean, E. Posner, S. Thomson, E. J. Wood, “The ethics of research with human participants and the value of reflexive openness” in *Oxford Handbook of Engaged Methodological Pluralism in Political Science*, J. M. Box-Steffensmeier, Ed. (Oxford University Press, 2024).

[89] D. Pager, R. Goldstein, H. Ho, B. Western, Criminalizing poverty: The consequences of court fees in a randomized experiment. Am. Sociol. Rev. **87**, 529–553 (2022).

[90] L. Nader, Up the Anthropologist: Perspectives Gained from Studying Up (Vintage Books, 1972), pp. 284–311.

[91] M. G. Findley, D. L. Nielson, J. Sharman, Global Shell Games: Experiments in Transnational Relations, Crime, and Terrorism (Cambridge University Press, 2014).

[92] R. A. Nielsen, “Ethics for experimental manipulation of religion” in *Ethics and Experiments: Problems and Solutions for Social Scientists and Policy Professionals*, S. Desposato Ed. (Routledge, 2016).

[93] M. S. Bernstein , Ethics and society review: Ethics reflection as a precondition to research funding. Proc. Natl. Acad. Sci. U.S.A. **118**, 1–8 (2021). 10.1073/pnas.2117261118 PMC8719852 34934006

[94] V. Eubanks, *Automating Inequality: How High-Tech Tools Profile, Police, and Punish the Poor* (St. Martin’s Press, 2018).

[95] Z. Obermeyer, B. Powers, C. Vogeli, S. Mullainathan, Dissecting racial bias in an algorithm used to manage the health of populations. Science **366**, 447–453 (2019). 31649194 10.1126/science.aax2342

[96] H. Baron, L. E. Young, From principles to practice: Methods to increase the transparency of research ethics in violent contexts. Polit. Sci. Res. Methods **10**, 1–8 (2021).

[97] K. Kao, M. R. Revkin, Retribution or reconciliation? Post-conflict attitudes toward enemy collaborators Am. J. Polit. Sci. **67**, 1–16 (2021).

[98] M. Findley, D. Nielson, “Obligated to deceive? Aliases, confederates, and the common rule in international field experiments” in *Ethics and Experiments: Problems and Solutions for Social Scientists and Policy Professionals*, S. Desposato Ed. (Routledge, 2016), pp. 151–170.

[99] J. Lyall, “Preregister Your Ethical Redlines: Vulnerable Populations, Policy Engagement, and the Perils of E-Hacking” in *Speaking Science to Power: Responsible Researchers and Policy Engagement*, R. A. Epstein, O. Kaplan, Eds. (Oxford University Press, Oxford, 2024).

[100] D. L. Teele, “Reflections on the ethics of field experiments” in *Field Experiments and Their Critics: Essays on the Uses and Abuses of Experimentation in the Social Sciences*, D. L. Teele, Ed. (Yale University Press, 2014), pp. 115–140.

[101] M. Ellsberg, L. Heise, Bearing witness: Ethics in domestic violence research. Lancet **359**, 1599–1604 (2002). 12047984 10.1016/S0140-6736(02)08521-5

[102] E. L. Paluck, D. P. Green, Deference, dissent, and dispute resolution: An experimental intervention using mass media to change norms and behavior in Rwanda. Am. Polit. Sci. Rev. **103**, 622–644 (2009).

[103] S. Baele, The ethics of new development economics: Is the experimental approach to development economics morally wrong? J. Philos. Econ. VII, 2–43 (2013).

[104] S. Hewlett, Consent to clinical research-adequately voluntary or substantially influenced? J. Med. Ethics **22**, 232–237 (1996). 8863149 10.1136/jme.22.4.232 PMC1377003

[105] M. Callen, M. Fajardo-Steinhauser, M. Findley, T. Ghani, Can digital aid deliver during humanitarian crises? arXiv [Preprint] (2024). https://arxiv.org/abs/2312.13432v3 (Accessed 22 July 2024).

[106] T. Broesch , Navigating cross-cultural research: Methodological and ethical considerations. Proc. R. Soc. B **287**, 20201245 (2020). 10.1098/rspb.2020.1245 PMC7542829 32962541

[107] D. W. Nickerson, S. D. Hyde, “Conducting research with ngos: Relevant counterfactuals from the perspective of subjects” in *Ethics and Experiments: Problems and Solutions for Social Scientists and Policy Professionals*, S. Desposato Ed. (Routledge, 2016), pp. 198–216.

[108] R. Khera, Some questions of ethics in randomized controlled trials. *Rev. Dev. Econ.* (2023).

[109] R. Epstein, O. Kaplan, “Conclusion: The future of responsible engagement” in *Speaking Science to Power: Responsible Researchers and Policymaking*, R. Epstein, O. Kaplan, Eds. (Oxford University Press, 2024).

[110] L. A. Fujii, Interviewing in Social Science Research: A Relational Approach (Routledge, 2018).

[111] A. Shesterinina, Humanising political violence: Lee Ann Fujii’s legacies for civil war studies. Civil Wars **25**, 577–588 (2023).

[112] W. Lin, D. P. Green, Standard operating procedures: A safety net for pre-analysis plans. PS Polit. Sci. Polit. **49**, 495–499 (2016).

[113] P. Shivayogi, Vulnerable population and methods for their safeguard. Perspect. Clin. Res. **4**, 53–57 (2013). 23533983 10.4103/2229-3485.106389 PMC3601707

[114] F. Ghosn, R. A. Nielsen, S. E. Parkinson, How to say “khalas” (American Political Science Association, 2021).

[115] A. C. Hartman, R. A. Blair, C. Blattman, Engineering informal institutions: Long-run impacts of alternative dispute resolution on violence and property rights in Liberia. J. Polit. **83**, 381–389 (2021).

[116] D. M. Butler, S. Desposato, Proposing a compensation requirement for audit studies. Polit. Stud. Rev. **20**, 147892992110529 (2021).

[117] R. Aguilar, “Ethical perspectives in countries without an institutional review board: The case of mexico” in *Ethics and Experiments: Problems and Solutions for Social Scientists and Policy Professionals*, S. Desposato Ed. (Routledge, 2016), pp. 139–148.

[118] J. B. Johnson, Protecting the community: Lessons from the Montana flyer project. PS Polit. Sci. Polit. **51**, 615–619 (2018).

[119] C. B. Barrett, M. R. Carter, Finding our balance? Revisiting the randomization revolution in development economics ten years further on. World Dev. **127**, 104789 (2020).

[120] R. K. Wilson, W. Mishler, J. Ishiyama, “Journal editors as ethics sheriffs” in *Ethics and Experiments: Problems and Solutions for Social Scientists and Policy Professionals*, S. Desposato Ed. (Routledge, 2016), pp. 262–266.

[121] E. J. Zechmeister, *Ethics and research in political science: The responsibilities of the researcher and the profession*, S. Desposato, Ed. (Routledge, 2016), pp. 269–275.

[122] R. A. Blair, M. Weintraub, Little evidence that military policing reduces crime or improves human security. Nat. Hum. Behav. **7**, 861–873 (2023). 37169936 10.1038/s41562-023-01600-1

[123] S. Loue, B. Loff, Is there a universal understanding of vulnerability? Experiences with Russian and Romanian trainees in research ethics. J. Empir. Res. Hum. Res. Ethics **8**, 17–27 (2013). 24384513 10.1525/jer.2013.8.5.17 PMC4090770

[124] S. Desposato, Subjects and scholars’ views on the ethics of political science field experiments. Perspect. Polit. **16**, 739–750 (2018).

[125] M. Costa, C. Crabtree, J. B. Holbein, M. Landgrave, Is that ethical? An exploration of political scientists’ views on research ethics. Res. Polit. **10**, 20531680231209553 (2023).

[126] O. O’neill, *Towards Justice and Virtue: A Constructive Account of Practical Reasoning* (Cambridge University Press, 1996).

[127] W. Rogers, M. M. Lange, Rethinking the vulnerability of minority populations in research. Am. J. Public Health **103**, 2141–2146 (2013). 24134375 10.2105/AJPH.2012.301200 PMC3828952

[128] E. Asiedu, D. Karlan, M. Lambon-Quayefio, C. Udry, A call for structured ethics appendices in social science papers. Proc. Natl. Acad. Sci. U.S.A. **118**, 1–10 (2021). 10.1073/pnas.2024570118 PMC8307908 34253610

[129] D. Haim, N. Ravanilla, R. Sexton, Sustained government engagement improves subsequent pandemic risk reporting in conflict zones. Am. Polit. Sci. Rev. **115**, 717–724 (2021).

[130] P. Bell, “The ethics of conducting psychiatric research in war-torn contexts” in *Researching Violently Divided Societies: Ethical and Methodological Issues*, M. Smyth, G. Robinson, Eds. (United Nations University Press, 2001), pp. 184–192.

[131] E. Pittaway, L. Bartolomei, R. Hugman, “Stop stealing our stories’’: The ethics of research with vulnerable groups. J. Hum. Rights Pract. **2**, 229–251 (2010).

[132] L. A. Fujii, Research ethics 101: Dilemmas and responsibilities. PS Polit. Sci. Polit. **45**, 717–723 (2012).

[133] E. L. Paluck, “Methods and ethics with research teams and ngos: Comparing experiences across the border of rwanda and democratic republic of congo” in *Surviving Field Research: Working in Violent and Difficult Situations*, J. C. King, C. L. Sriram, J. A. Mertus, O. Martin-Ortega, J. Herman Eds. (Routledge, 2009), pp. 38–56.

[134] S. P. Campbell, Ethics of research in conflict environments. J. Glob. Sec. Stud. **2**, 89–101 (2017).

[135] K. Cronin-Furman, M. Lake, Ethics abroad: Fieldwork in fragile and violent contexts. PS Polit. Sci. Polit. **51**, 607–614 (2018).

[136] E. Dauphinée, The Ethics of Researching War: Looking for Bosnia (Manchester University Press, 2007).

[137] K. D. Bond, Reflexivity and revelation. Qualit. Multi-Method Res. **16**, 45–47 (2018).

[138] L. M. MacLean, E. Posner, S. Thomson, E. J. Wood, “Research ethics and human subjects: A reflexive openness approach” in *American Political Science Association Organized Section for Qualitative and Multi-Method Research, Qualitative Transparency Deliberations, Working Group Final Reports, Report I.2* (August 2018). 10.2139/ssrn.3332887. Deposited 15 February 2019.

[139] M. G. Findley, F. Ghosn, S. J. Lowe, “Replication Data for: Vulnerability in Research Ethics: A Call for Assessing Vulnerability and Implementing Protections”. Harvard Dataverse, V1. 10.7910/DVN/FC5IPF. Deposited 18 July 2024. PMC11348164 39141349

