# Supplementary material for: Vulnerability in research ethics: A call for assessing vulnerability and implementing protections
Source: Proc Natl Acad Sci U S A. 2024 Aug 14;121(34):e2322821121. doi: 10.1073/pnas.2322821121 (PMC11348164; doi:10.1073/pnas.2322821121)
Supplement: Supplementary file 1 — Appendix 01 (PDF) [file pnas.2322821121.sapp.pdf]

Supplementary Information for

Vulnerability in research ethics: A call for assessing vulnerability and implementing protections

Michael G. Findley, Faten Ghosn, and Sara Lowe

Corresponding Author: Michael Findley
E-mail: mikefindley@utexas.edu

This PDF file includes:

Supplementary text

Supporting Information Text

Full Coding Process

Sampling Frame. The sampling frame for the data was the 2019 International Compilation of Human Research Standards, which is a document updated annually by the Department of Health and Human Services (HHS). It contained a list of over 1,000 human subjects research standards for 131 countries covering both medical and general social-behavioral standards. 92 of these countries had GSB regulations and we identified an additional 15 countries with GSB regulations. Our sample includes 107 countries with 355 GSB documents in total.

Documents. The documents are categorized by the research areas that are targeted and include: General (or applicable to most types of research), Drugs and Devices, Clinical Trial Registries, Research Injury, Social-Behavioral Research, Privacy/Data Protection, Human Biological Materials, Genetic, and Embryos, Stem Cells, and Cloning. We recorded meta-information for all documents including, titles, years of publication, country of origin, year of origin, and document type. For the General and Social-Behavioral areas, we coded the vulnerability categories. The documents can also be classified by whether they are Regulations, Legislation, or Guidelines. All documents, regardless of this latter classification, were coded.

Coding Process. To gather information about vulnerability classifications in General and Social-Behavioral (GSB) documents, our research team reviewed each of these documents and coded the words associated with groups classified as "vulnerable." This list of words started with basic vulnerabilities mentioned in widely used human subject research documents, such as the *Common Rule* (45 CFR 46), the *Belmont Report*, and the *Declaration of Helsinki*, and then developed inductively (with backcoding of all previous documents). The initial list of words included prisoners, pregnant women, and children, because of their centrality in traditional documents. As the team coded more documents and identified new categories, the list expanded. To find these words and decide whether they were classifying a group or condition as vulnerable, the students utilized a machine-learning algorithm that, when given a list of indicators, scanned documents and produced a "Synopsis," or a collection of every mention of every word and four lines of context above and below the mention of the word. The team generated synopses and manually read every machine-identified mention to decide whether the authors of the document were classifying a certain group or condition as "vulnerable" in human subjects research.

Classifying Mentions of Vulnerability. Many of these documents explicitly named certain groups as vulnerable ("vulnerable groups such as prisoners, pregnant women, and children"). Another common way we discerned classifications of vulnerability was through the discussion of "protections" for certain specific groups. The documents did not always explicitly use the term 'vulnerable' when naming a vulnerable group, so research assistants utilized context in coding a group or characteristic as vulnerable. We are thus confident that the coding is not dependent on linguistic idiosyncracies, and instead captures the underlying construct.

Codifying Common Documents. A document was considered to be 'common' when it was frequently referenced by other, separate documents. For example, a document might name its own classifications of vulnerable groups *and* also mention its adoption of the *Belmont Report*, the *International Ethical Guidelines for Health-Related Research Involving Humans from the Council of International Organizations for Medical Sciences* (CIOMS), or the *Common Rule*, among others. If a specific document from a government agency mentioned the adoption of a common document, we coded the vulnerability categories of the common documents for the relevant specific documents. For example, if a Belgian research document named women, fetuses, and refugees as vulnerable but also adopted the *Belmont Report*, it would be coded as having classified as vulnerable women, fetuses, and refugees, as well as prisoners and minors.

Country Selection and Missing Countries. We included all countries with general research or social-behavioral (GSB) documents from the broader set of countries with ethics documents. The vast majority of documents and mentions of vulnerability were taken from documents found in the *International Compilation of Human Research Standards*. We cross-referenced the UN Member State list with the HHS *Compilation*, and when a country was not included in the *Compilation*, we researched it individually to confirm that it did not, in fact, have GSB documents with mentions of vulnerability. In nearly all cases, we could not find any documents with human subjects research protections. However, in 15 countries that do not appear in the *Compilation* (Afghanistan, Bahrain, Cape Verde, Cook Islands, Guam, Hong Kong, Lebanon, Lesotho, Niger, Oman, Papua New Guinea, Saint Helena, Samoa, Seychelles, and Swaziland), we found relevant GSB documents sometimes with vulnerability mentions. For those cases, we included the documents and categories in the data set. With additional research we also found documents in non-UN Member States (Bermuda, Cook Islands, Guam, Hong Kong, Saint Helena, Swaziland, and Taiwan). The countries that are not included do not have official research legislation, regulation, or guidelines that refer specifically to general or social-behavioral research, or they have none that can be accessed publicly online.

Replication Files. Replication files for version 1.0 of the data are available on Dataverse at [link here].

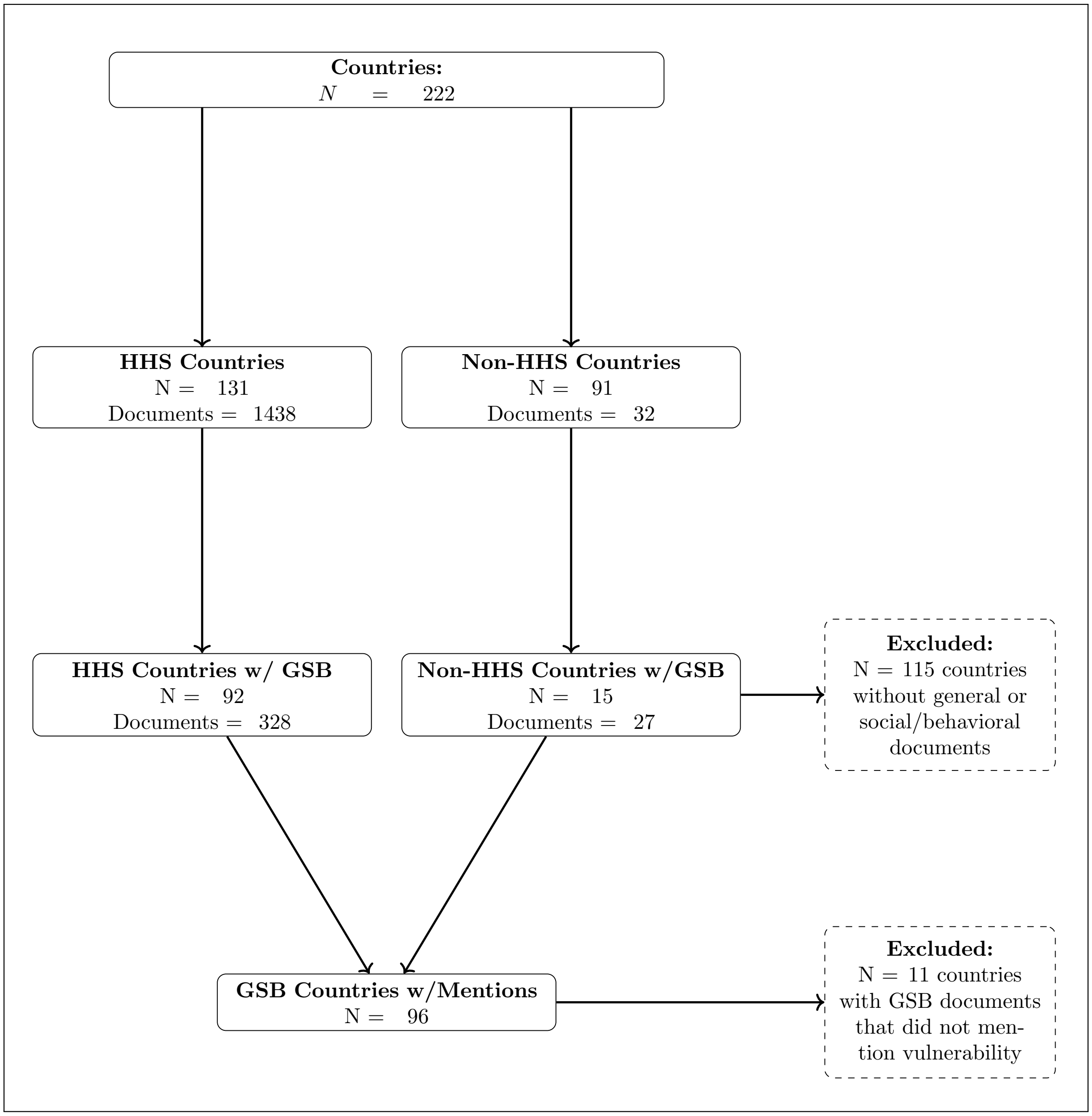

Fig. S1. Coding Summary

Notes: Summary of selection of countries/documents for coding. Note that the country counts include the EU as a separate entity.

Frequencies, Characteristics, Recommendations

Table S1. Complete List of Vulnerability Mentions

Category	Mentions	Category	Mentions	Category	Mentions
Child	92	Public Emergency	23	Stateless Persons	5
Age	74	Laboratory Staff	22	Ease of Access	5
Minor	67	HIV/AIDS	22	Structural Barriers	5
Mentally Disabled/Ill	66	Of Childbearing Age/Fertile	22	LGBTQ+ Status	5
Pregnant	62	Restricted Capacity	22	Pharmaceutical Industry Employees	5
Mentally Incapacitated	51	Indigenous	21	Fathers	5
Economic/Poverty	50	Ethnic Minority	21	Single Mothers	5
Education	49	Restricted Liberty	21	Developing Country	5
Impaired Autonomy	49	Displaced	20	Prosecuted	4
Physically Disabled	48	Marginalized Groups	20	Cadavers/Deceased Persons	4
Relationship to Authority	48	Threat of Stigma	20	Substance Use	4
Access to social goods	45	Diseased	20	Participant in Control Group	4
Cognitive Impairment	45	Student	19	Internally Displaced Persons (IDPs)	3
Undue Influence	45	Parents	19	Captive/Exiled Pop	3
Dependent	44	Victim of Abuse	19	Migrant	3
Fetus/Neonate/unborn child	42	Manipulable	19	Persecuted	3
Women	41	Access to Healthcare	18	In Child Protective Services	3
Incapacitated	41	Breastfeeding	18	Trade Union Membership	3
Linguistic Proficiency	40	Sex Workers	17	Single Parents	3
Racial Minority	39	Threat of Violence	17	Immuno-compromised	3
Incarcerated	37	Decisionally Impaired	17	Intoxication	3
Institutionalized	37	Asylum Seeker	16	Rural and Remote Communities	3
Infant	36	Trafficked Persons	16	Victims of a Crime	2
Criminal Convictions	35	Politically Powerless	16	People in Detention Centers	2
Political Affiliation	35	Women seeking an illegal abortion	16	Threat of Privation	2
Terminally Ill	35	Medical/Nursing Student	16	Access to Information	2
Diminished Autonomy	35	Physical Frailty	16	Precarious Housing	2
Physically Ill	34	Unfamiliar w/ Modern Medical Concepts	16	Widows	2
Literacy	31	Living in an Authoritarian Environment	16	Skin Color	2
Welfare Recipient	31	Socially Weak/Disadvantaged	15	Public Official	2
Presence of Coercion	30	Healthy People	15	Informal/Unstable Occupation	2
Refugee Status	29	Comatose	15	Inventors/Intellectual Rights	2
Gender	29	War Criminal	14	Degenerative Conditions	2
Ethnicity	29	Property Ownership	14	Whistleblowers	2
Ill	29	Non-existent Autonomy	14	Prisoner of War	1
Embryo	29	Other Country	14	Ex Combatants	1
Cultural Differences	29	Genetic Heritage	13	Loss of Family in War/Conflict	1
Soldiers	28	Attempting Pregnancy	13	No Social Security Coverage	1
Religion	28	Drug Dependence	12	Persons with Care Obligations	1
Motherhood/Family	28	Criminal	11	In Mourning	1
Elderly	27	Marital Position	11	Divorcees	1
Homeless Persons	27	Unconscious People	11	Students with Children	1
Unemployment	27	Alcoholism	9	International Students	1
Drug Usage	26	Immigrants	8	Food Insecurity	1
Illegal Activity	25	Breastfeeding Children	8	Neurological Disorders	1
In Nursing Home	25	Orphans	8	Sexually Transmitted Diseases	1
Patients in Emergency Situations	25	Hospitalized Patients	8	Sensory Impairment	1
Nomads	24	Stem Cells	8	Visual Impairment	1
Employed Persons	24	Philosophical Differences	7	Addiction	1
Mothers	24	Injured	6	Victims of Data Security Breaches	1
Police Officer	23	Natural Hazards	6	Foreign Nationals	1

Notes: All original 153 categories sorted by number of mentions (reported in second, fourth, and six columns).

Table S2. Consolidated List of Vulnerability Mentions.

Category	Mentions	Category	Mentions
Person with Inherently Decreased Autonomy	223	Located in Developing or Disadvantaged Region	24
Youth/Minor	214	Public Emergency	23
Cognitive or Mental Impairment	163	HIV/AIDS	22
Person with Outside Forces Decreasing Autonomy	162	Indigenous	21
Direct Involvement in Criminal Activities	114	Threat of Stigma	20
Motherhood	97	Victim of Abuse	19
Employees	93	Threat of Violence	17
Human Rights	92	Members of Communities Unfamiliar w/ Modern Medical Concepts	16
Minority (Racial/Ethnic)	91	Women Seeking Abortion	16
Physically Unwell	88	Healthy People	15
Education Level	80	Socially Weak/Disadvantaged	15
Access to Social Safety Net	76	Marital Status	14
Age	74	Property Owners	14
Preborn Child	71	Attempting Pregnancy	13
Gender	70	Genetic Heritage	13
Forcibly Displaced	68	Voluntary Migration	11
Physical Disability or Handicap	64	Stem Cells	8
Pregnant	62	Injured	6
Elderly/Old Age	52	Natural Hazards	6
Usage or Addiction to Substance	52	LGBTQ+ Status	5
Economic/Poverty	50	Cadavers/Deceased Persons	4
Armed Conflict	45	Participant in Control Group	4
Difference in Cultural or National Identity	44	Intoxication	3
Linguistic Proficiency	40	Trade Union Membership	3
Institutionalized	37	Detained	2
Student	37	Investors/Intellectual Rights	2
Philosophical or Religious Differences	35	Sensory Impairment	2
Terminally Ill	35	Whistleblowers	2
Patients	33	Caretaker	1
Citizenship	32	Food Insecurity	1
Welfare Recipient	31	In Mourning	1
Insecure Housing	29	Sexually Transmitted Diseases	1
Insecure Occupation	29	Victims of Data Security Breaches	1
Parenthood	27		
Unconscious/Comatose	26		

Notes: Consolidated categories with number of mentions in second and fourth columns.

Table S3. Categories Organized by Levels of Aggregation.

Type	Broader Category	Vulnerability	Type	Broader Category	Vulnerability
Political	Criminal	Criminal	Social	Parenthood	Fathers
		Criminal Convictions			Single Parents
		Illegal Activity	Social	Property Owners	Property Ownership
		People Under Arrest	Social	Socially Weak/Disadvantage	Socially Weak/Disadvantaged
		Prosecuted	Social	Religious/Spiritual Beliefs	Philosophical Differences
		Incarcerated			Religion
		Victims Of A Crime	Social	Threat Of Stigma	Threat Of Stigma
Political	Armed Conflict	War Criminal			Threat Of Violence
		Prisoner Of War	Social	Victim Of Abuse	Victim Of Abuse
		Recruitment By Extremist Groups	Social	Pet Owners	Pet Owners
		Ex Combatants	Economic	Economic/Poverty	Economic/Poverty
		Soldiers	Economic	Food Insecurity	Food Insecurity
		Loss Of Family In War/Conflict	Economic	Investors/Intellectual Rights	Inventors/ Intellectual Rights
Political	Detained	People In Detention Centers	Economic	Welfare Recipient	Welfare Recipient
Political	Displaced	Displaced	Health	Cadavers/Deceased Persons	Cadavers/Deceased Persons
		Internally Displaced Persons	Health	Cognitive/Mental Impairment	Cognitive Impairment
		Refugee Status			Mentally Disabled/Ill
		Asylum Seeker			Mentally Incapacitated
Political	Citizenship	Stateless Persons			Neurological Disorders
		Nomads	Health	Healthy People	Healthy People
		Captive/Exiled Pop	Health	Hiv/Aids	Hiv/Aids
Political	Migrants	Immigrants	Health	Ill	Diseased
		Migrant			Ill
Political	Indigenous	Indigenous			Immuno-Compromised
Political	Human Rights	Persecuted			Physically Ill
		Marginalized Groups			Degenerative Conditions
		Threat Of Privation			Terminally Ill
		Trafficked Persons			Sexually Transmitted Diseases
		Politically Powerless	Health	Injured	Injured
Political	Political Affiliation	Political Affiliation	Health	Motherhood	Mothers
Social	Abortion	Women Seeking Illegal Abortion			Of Childbearing Age/Fertile
Social	Access To Social Goods	Access To Healthcare			Single Mothers
		Access To Information			Breastfeeding
		Access To Social Goods			Motherhood/Family
		Ease Of Access	Health	Patients	Forensic Patients
		No Social Security Coverage			Patients In Emergency Situations
		Structural Barriers			Hospitalized Patients
Social	Age	Age	Health	Physical Disability/Handicap	Physical Frailty
		Child			Physically Disabled
		Minor	Health	Pregnancy	Pregnant
		Infant			Attempting Pregnancy
		Breastfeeding Children	Health	Sensory Impairment	Sensory Impairemnt
		In Child Protective Services			Visual Impairemnt
		Orphans	Health	Stem Cells	Stem Cells
		Elderly	Health	Substance Use	Addiction
		In Nursing Home			Substance Use
Social	Caretaker	Persons With Care Obligations			Alcoholism
Social	Education	Education			Drug Dependence
		Literacy			Drug Usage
Social	Gender	Gender			Intoxication
		Women	Health	Unconscious/Comatose	Comatose
Social	Homeless	Precarious Housing			Unconscious People
		Homeless Persons	Health	Preborn Child	Fetus/Neonate/Unborn Child
Social	In Mourning	In Mourning			Embryo
Social	Institutionalized	Institutionalized	Health	Linguistic Proficiency	Linguistic Proficiency
Social	Lgbtq+ Status	Lgbtq+ Status	Health	Unfamiliar Medical Concepts	Unfamiliar Modern Medical Concepts
...	...	...	...	...	...

Notes: Columns 1 and 4 (Type) correspond to the innermost circle of Manuscript Figure 1. Columns 2 and 5 (Broader Category) correspond to the inner ring. Columns 3 and 6 correspond to the original 153 categories before condensing to the list of 68. (See next page for continuation of the table.)

Table S4. Categories Organized by Levels of Aggregation (cont).

Type	Broader Category	Vulnerability	Type	Broader Category	Vulnerability
Social	Marital Status	Marital Position	General	Control Group	Participant In Control Group
		Divorcees	General	Emergency Situations	Natural Hazards
		Widows			Public Emergency
Social	Minority (Racial/Ethnic)	Ethnic Minority	General	Impaired Autonomy	Undue Influence
		Ethnicity			Diminished Autonomy
		Racial Minority			Impaired Autonomy
		Skin Color			Decisionally Impaired
Social	Genetic Heritage	Genetic Heritage			Incapacitated
Social	Occupation	Employed Persons			Restricted Capacity
		Pharmaceutical Employees			Non-Existent Autonomy
		Laboratory Staff			Dependent
		Police Officer			Presence Of Coercion
		Public Official			Manipulable
		Sex Workers			Relationship To Authority
		Student			Restricted Liberty
		Students With Children	General	Information Breach	Victims Of Data Security Breaches
		International Students			Whistleblowers
		Medical/Nursing Student	General	Place Of Origin	Cultural Differences
		Informal/Unstable Occupation			Other Country
		Unemployment			Foreign Nationals
		Trade Union Membership			Developing Country
Social	Parenthood	Parents			Authoritarian Environment
					Rural And Remote Communities
					Ecologically Unfavorable Regions

Notes: Columns 1 and 4 (Type) correspond to the innermost circle of Manuscript Figure 1. Columns 2 and 5 (Broader Category) correspond to the inner ring. Columns 3 and 6 correspond to the original 153 categories before condensing to the list of 68. (See prior page for beginning of the table.)

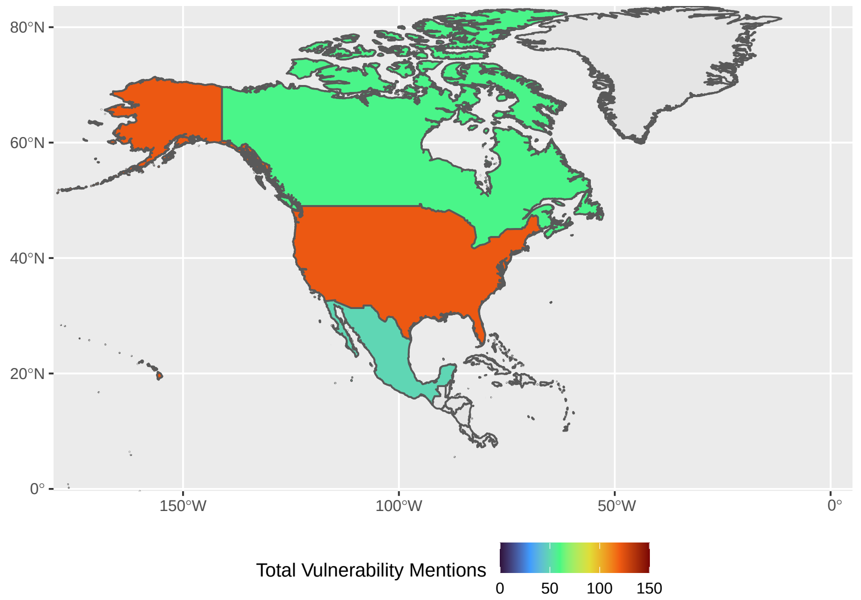

Fig. S2. Number of Unique Vulnerability Mentions by Country in North America and the Caribbean
Notes: See Table S5 for corresponding overall and sectoral mentions.

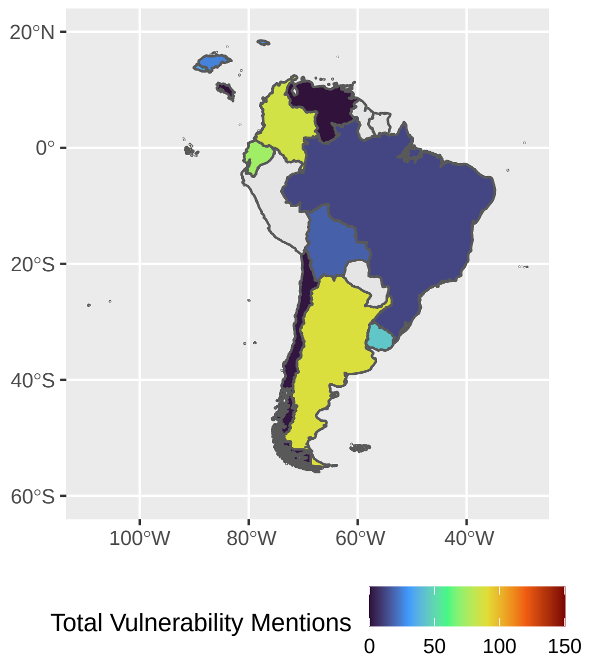

Fig. S3. Number of Unique Vulnerability Mentions by Country in South America
Notes: See Table S5 for corresponding overall and sectoral mentions.

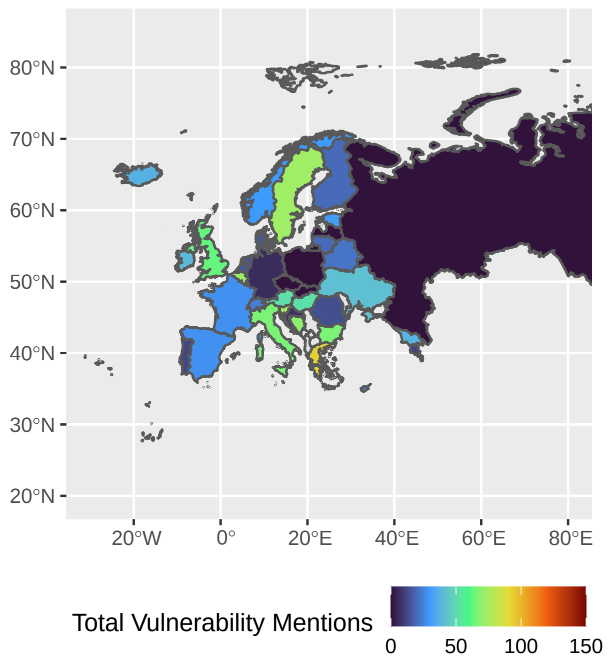

Fig. S4. Number of Unique Vulnerability Mentions by Country in Europe
Notes: See Table S6 for corresponding overall and sectoral mentions.

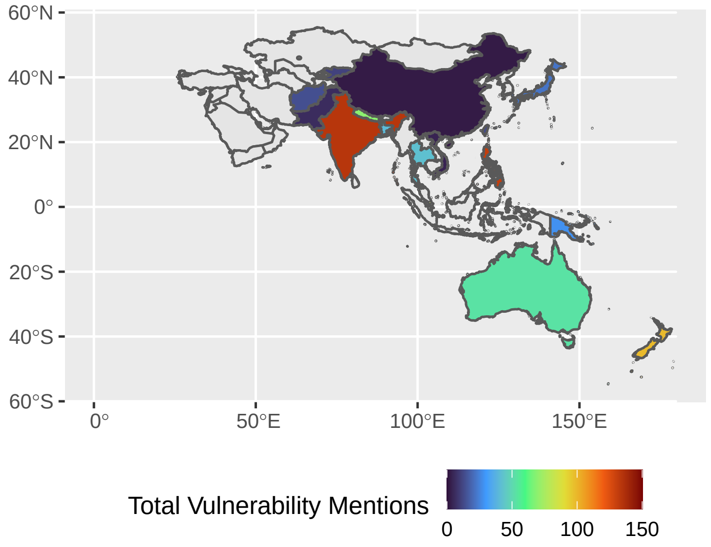

Fig. S5. Number of Unique Vulnerability Mentions by Country in Asia/Pacific
Notes: See Table S7 for corresponding overall and sectoral mentions.

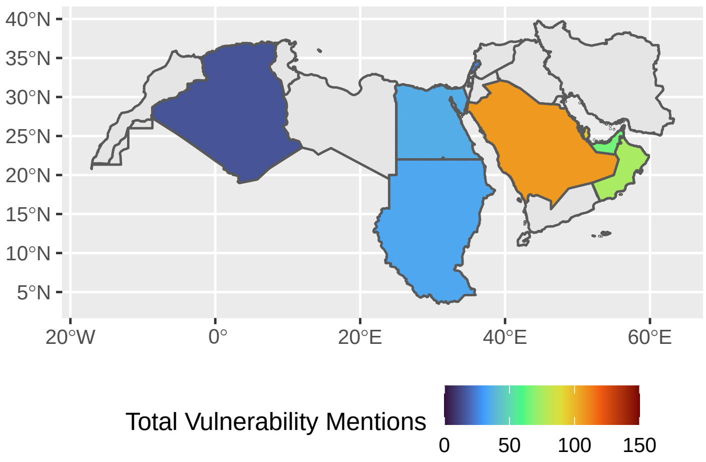

Fig. S6. Number of Unique Vulnerability Mentions by Country in the Middle East
Notes: See Table S8 for corresponding overall and sectoral mentions.

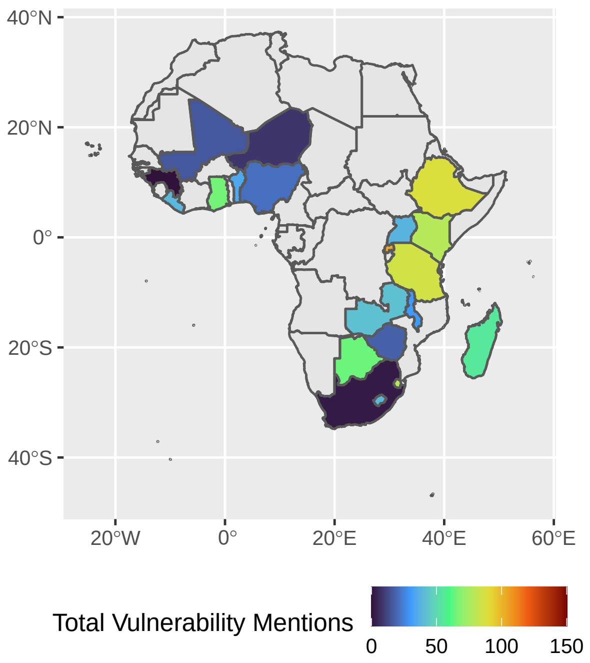

Fig. S7. Number of Unique Vulnerability Mentions by Country in Sub-Saharan Africa
Notes: See Table S9 for corresponding overall and sectoral mentions.

Table S5. Number of *Overall* and *Sectoral* Mentions for 46 Countries in the Americas and the Caribbean

Country	Overall	Political	Health	Social	Economic	Other
United States	121	17	23	65	1	15
Argentina	88	12	13	51	1	11
Colombia	85	13	15	45	1	11
Bermuda	73	12	12	40	1	8
Ecuador	73	9	14	43	1	6
Canada	59	8	11	29	1	10
Mexico	49	3	11	28	1	6
Uruguay	44	4	8	26	0	6
El Salvador	34	5	4	22	0	3
Jamaica	29	2	3	20	0	4
Honduras	25	5	6	12	0	2
Bolivia	18	1	5	12	0	0
Trinidad and Tobago	15	2	2	9	0	2
Brazil	12	1	2	8	0	1
Chile	1	0	0	1	0	0
Barbados	0	0	0	0	0	0
Costa Rica	0	0	0	0	0	0
Cuba	0	0	0	0	0	0
Dominica	0	0	0	0	0	0
Dominican Republic	0	0	0	0	0	0
Grenada	0	0	0	0	0	0
Guatemala	0	0	0	0	0	0
Guyana	0	0	0	0	0	0
Haiti	0	0	0	0	0	0
Nicaragua	0	0	0	0	0	0
Panama	0	0	0	0	0	0
Paraguay	0	0	0	0	0	0
Peru	0	0	0	0	0	0
Venezuela	0	0	0	0	0	0
Antigua and Barbuda	0	0	0	0	0	0
Aruba	0	0	0	0	0	0
The Bahamas	0	0	0	0	0	0
Belize	0	0	0	0	0	0
British Virgin Islands	0	0	0	0	0	0
Cayman Islands	0	0	0	0	0	0
Curacao	0	0	0	0	0	0
Greenland	0	0	0	0	0	0
Puerto Rico	0	0	0	0	0	0
St. Kitts and Nevis	0	0	0	0	0	0
St. Lucia	0	0	0	0	0	0
St. Martin	0	0	0	0	0	0
St. Pierre and Miquelon	0	0	0	0	0	0
St. Vincent and the Grenadines	0	0	0	0	0	0
Suriname	0	0	0	0	0	0
Turks and Caicos Islands	0	0	0	0	0	0
Virgin Islands	0	0	0	0	0	0
Totals	726	94	129	411	7	85

$Notes$: All categories reported with number of mentions in columns 2–7. Countries below the midrule line did not appear in the HHS list. Countries above the midrule line but with 0s in columns 2–7 appeared in the HHS list but contained human subjects research documents for medical, stem cell, data protection, or other non-general or non-social-behavioral documents.

Table S6. Number of *Overall* and *Sectoral* Mentions for 56 Countries (and EU) in Europe and Central Asia

Country	Overall	Political	Health	Social	Economic	Other
European Union	130	18	24	72	1	15
Greece	93	14	15	53	1	10
Slovenia	73	1	18	44	0	10
Sweden	73	12	11	40	1	9
Belgium	72	5	13	42	1	11
Bosnia and Herzegovina	69	4	16	38	1	10
Bulgaria	66	8	11	41	1	5
Italy	66	11	12	38	1	4
United Kingdom	63	3	13	37	1	9
Austria	52	6	13	30	1	2
Hungary	52	1	13	28	1	9
Ukraine	42	8	7	24	1	2
Ireland	40	3	7	22	0	8
Georgia	38	0	9	22	1	6
Iceland	37	2	5	22	0	8
Estonia	31	4	2	20	1	4
Norway	30	2	6	20	1	1
France	28	0	5	16	1	6
Spain	28	0	8	16	0	4
Cyprus	24	2	5	15	1	1
Switzerland	24	5	4	13	0	2
Belarus	22	4	5	12	0	1
Finland	20	0	3	15	0	2
Lithuania	20	1	5	12	0	2
Denmark	16	0	3	11	0	2
Netherlands	16	2	1	9	1	3
Romania	14	0	5	9	0	0
Portugal	12	0	4	8	0	0
Armenia	11	2	0	6	0	3
Croatia	7	1	0	6	0	0
Germany	6	1	0	5	0	0
Czech Republic	0	0	0	0	0	0
Kazakhstan	0	0	0	0	0	0
Latvia	0	0	0	0	0	0
Luxembourg	0	0	0	0	0	0
Moldova	0	0	0	0	0	0
Montenegro	0	0	0	0	0	0
North Macedonia	0	0	0	0	0	0
Poland	0	0	0	0	0	0
Russia	0	0	0	0	0	0
San Marino	0	0	0	0	0	0
Serbia	0	0	0	0	0	0
Slovakia	0	0	0	0	0	0
Tajikistan	0	0	0	0	0	0
Turkey	0	0	0	0	0	0
Uzbekistan	0	0	0	0	0	0
Albania	0	0	0	0	0	0
Andorra	0	0	0	0	0	0
Azerbaijan	0	0	0	0	0	0
Channel Islands	0	0	0	0	0	0
Faroe Islands	0	0	0	0	0	0
Gibraltar	0	0	0	0	0	0
Isle of Man	0	0	0	0	0	0
Kosovo	0	0	0	0	0	0
Lichtenstein	0	0	0	0	0	0
Monaco	0	0	0	0	0	0
Turkmenistan	0	0	0	0	0	0
Totals	1275	120	243	746	17	149

Notes: All categories reported with number of mentions in columns 2–7. Countries below the midrule line did not appear in the HHS list. Countries above the midrule line but with 0s in columns 2–7 appeared in the HHS list but contained human subjects research documents for medical, stem cell, data protection, or other non-general or non-social-behavioral documents.

Table S7. Number of *Overall* and *Sectoral* Mentions for 48 Countries in Asia & the Pacific

Country	Overall	Political	Health	Social	Economic	Other
India	134	18	25	74	1	16
Philippines	131	25	19	71	2	14
New Zealand	99	17	16	56	1	9
Singapore	82	8	14	47	1	12
Nepal	69	5	12	40	1	11
Australia	53	11	6	27	0	9
Thailand	42	3	11	24	0	4
Bangladesh	40	5	7	22	1	5
Samoa	32	1	7	19	0	5
Papua New Guinea	28	3	5	15	1	4
Japan	22	0	4	13	1	4
Guam	21	1	2	13	0	5
Hong Kong	16	2	1	10	0	3
Afghanistan	14	0	3	10	1	0
Taiwan	13	2	0	6	0	5
Kyrgyzstan	11	2	0	6	0	3
Pakistan	6	0	2	4	0	0
Viet Nam	4	0	0	4	0	0
China	2	0	0	1	0	1
Indonesia	0	0	0	0	0	0
Malaysia	0	0	0	0	0	0
Myanmar	0	0	0	0	0	0
South Korea	0	0	0	0	0	0
Sri Lanka	0	0	0	0	0	0
American Samoa	0	0	0	0	0	0
Bhutan	0	0	0	0	0	0
Brunei	0	0	0	0	0	0
Cambodia	0	0	0	0	0	0
Cook Islands	0	0	0	0	0	0
Fiji	0	0	0	0	0	0
French Polynesia	0	0	0	0	0	0
Kiribati	0	0	0	0	0	0
Lao PDR	0	0	0	0	0	0
Macao SAR	0	0	0	0	0	0
Maldives	0	0	0	0	0	0
Marshall Islands	0	0	0	0	0	0
Micronesia	0	0	0	0	0	0
Mongolia	0	0	0	0	0	0
Nauru	0	0	0	0	0	0
New Caledonia	0	0	0	0	0	0
North Korea	0	0	0	0	0	0
Northern Mariana Islands	0	0	0	0	0	0
Palau	0	0	0	0	0	0
Solomon Islands	0	0	0	0	0	0
Timor-Leste	0	0	0	0	0	0
Tonga	0	0	0	0	0	0
Tuvalu	0	0	0	0	0	0
Vanuatu	0	0	0	0	0	0
Totals	819	103	134	462	10	110

$Notes$: All categories reported with number of mentions in columns 2–7. Countries below the midrule line did not appear in the HHS list. Countries above the midrule line but with 0s in columns 2–7 appeared in the HHS list but contained human subjects research documents for medical, stem cell, data protection, or other non-general or non-social-behavioral documents.

Table S8. Number of *Overall* and *Sectoral* Mentions for 23 Countries in the Middle East and North Africa

Country	Overall	Political	Health	Social	Economic	Other
Saudi Arabia	107	16	19	58	1	13
Qatar	96	16	14	53	1	12
Oman	75	9	14	41	1	10
United Arab Emirates	65	7	10	37	1	10
Egypt	36	4	5	22	1	4
Bahrain	34	3	6	19	1	5
Sudan	34	1	7	20	0	6
Lebanon	25	4	5	14	0	2
Algeria	15	0	6	9	0	0
Iran	0	0	0	0	0	0
Israel	0	0	0	0	0	0
Jordan	0	0	0	0	0	0
Kuwait	0	0	0	0	0	0
Malta	0	0	0	0	0	0
Tunisia	0	0	0	0	0	0
Djibouti	0	0	0	0	0	0
Iraq	0	0	0	0	0	0
Libya	0	0	0	0	0	0
Morocco	0	0	0	0	0	0
Syrian Arab Republic	0	0	0	0	0	0
West Bank and Gaza	0	0	0	0	0	0
Yemen	0	0	0	0	0	0
Totals	487	60	86	273	6	62

$Notes$: All categories reported with number of mentions in columns 2–7. Countries below the midrule line did not appear in the HHS list. Countries above the midrule line but with 0s in columns 2–7 appeared in the HHS list but contained human subjects research documents for medical, stem cell, data protection, or other non-general or non-social-behavioral documents.

Table S9. Number of *Overall* and *Sectoral* Mentions for 48 Countries in Sub-Saharan Africa

Country	Overall	Political	Health	Social	Economic	Other
Rwanda	104	0	52	52	0	0
Ethiopia	88	16	12	47	1	12
United Republic of Tanzania	85	14	16	48	1	6
Kenya	78	8	16	46	0	8
Swaziland	78	13	12	41	1	11
Ghana	65	12	12	36	1	4
Botswana	64	7	11	37	1	8
Madagascar	55	5	13	30	1	6
Zambia	42	6	8	26	1	1
Lesotho	40	2	12	24	1	1
Liberia	39	4	9	23	1	2
Uganda	38	2	5	20	1	10
Benin	34	1	8	21	0	4
Gambia	34	1	10	21	1	1
Malawi	30	3	4	19	1	3
Nigeria	21	2	4	12	0	3
Zimbabwe	18	2	1	15	0	0
Mali	16	1	5	9	0	1
Niger	8	1	1	5	1	0
Seychelles	4	0	1	2	0	1
Saint Helena	2	0	0	1	0	1
South Africa	2	0	1	1	0	0
Burkina Faso	0	0	0	0	0	0
Cameroon	0	0	0	0	0	0
Côte d'Ivoire	0	0	0	0	0	0
Democratic Republic of the Congo	0	0	0	0	0	0
Guinea	0	0	0	0	0	0
Mozambique	0	0	0	0	0	0
Senegal	0	0	0	0	0	0
Sierra Leone	0	0	0	0	0	0
Angola	0	0	0	0	0	0
Burundi	0	0	0	0	0	0
Cape Verde	0	0	0	0	0	0
Central African Republic	0	0	0	0	0	0
Chad	0	0	0	0	0	0
Comoros	0	0	0	0	0	0
Equatorial Guinea	0	0	0	0	0	0
Eritrea	0	0	0	0	0	0
Gabon	0	0	0	0	0	0
Guinea-Bissau	0	0	0	0	0	0
Mauritania	0	0	0	0	0	0
Mauritius	0	0	0	0	0	0
Namibia	0	0	0	0	0	0
Republic of the Congo	0	0	0	0	0	0
São Tomé and Principe	0	0	0	0	0	0
Somalia	0	0	0	0	0	0
South Sudan	0	0	0	0	0	0
Togo	0	0	0	0	0	0
Totals	945	100	213	536	13	83

Notes: All categories reported with number of mentions in columns 2–7. Countries below the midrule line did not appear in the HHS list. Countries above the midrule line but with 0s in columns 2–7 appeared in the HHS list but contained human subjects research documents for medical, stem cell, data protection, or other non-general or non-social-behavioral documents.

Table S10. Characteristics of Example Vulnerability Categories

Characteristics	Examples
Inherent	
-Do individuals/groups have limited freedom to consent?	Cognitive impairment
-Do individuals/groups have limited capacity to consent?	Mental disability
-Are individuals/groups at a non-suitable developmental stage?	Non-adults; elderly
-Are individuals/groups at greater risk of undue influence or coercion?	Illiterate
-Have individuals suffered abuse that could be retriggered?	Abused
-Do individuals suffer from mental illness that could worsen?	Mental illness
-Do language barriers prevent some individuals from accessing research benefits?	Immigrants
-Are individuals/groups more likely to acquire other inherent characteristics?	Elderly; New physical/mental illness
Situational	
-Are individuals/groups susceptible to undue influence in unequal situations?	Lower castes/classes
-Do cultural norms condition submission and toleration?	Gender
-Are individuals/groups in temporary circumstances?	Displaced; Post-disaster
-Are individuals/groups repeatedly exploited, or susceptible to exploitation?	Politically unrecognized groups
-Do individuals/groups lack the ability to safeguard their own interests in research?	Institutionalized
-Are individuals/groups difficult to reach such that policies/research are not extended?	Conflict zones
-Do situational factors compound possible harms to individuals/groups?	Conflict/displacement
Induced	
-Is an intervention untested or early-stage?	Social pressure
-Is there consensus in the field/society that the intervention will not induce/exacerbate vulnerability?	Information primes
-Is an intervention always (individually) risky?	Misinformation
-Is an intervention always directed at certain individuals/groups?	Displaced
-Can the negative effects of an intervention spillover beyond direct subjects?	Community-driven development
-Do research participants disclose unrelated, but sensitive material in the course of research?	Interview where disclosure possible
-Are proposed benefits unrealistic for some individuals/groups?	Terminal care; authoritarian environments
-Are risks overemphasized thereby excluding those with inherent/situational vulnerabilities?	Therapeutic complements to social interventions
-Has research unfairly or inequitably targeted some individuals/groups historically?	Prisoners; racial/ethnic minorities

Table S11. Who is Responsible? And How?

Stakeholders	Incentives and Norms
-Researchers	Researchers should prioritize implementation of a TAPIR process for addressing vulnerability.
-Ethics boards	Prioritize research-induced vulnerability over categories and characteristics.
	Incorporate global vulnerability categories and characteristics.
	Engage experts to evaluate and encourage a more robust TAPIR process.
-Associations/editors/reviewers	Associations and editors should require disclosure or include badges for TAPIR discussion.
	Reviewers should ask for the inclusion of ethics discussions.
	Editors and reviewers might first focus on inclusion of any discussion, and address content as training develops.
-Research/policy community	Hold forums for discussion of vulnerability and ethics, including moderated confidential forums.
	Highlight and reward positive ethics practices while also developing accountability mechanisms.
	Incorporate ethics more centrally into graduate education.
	Create spaces for posting public ethics plans, similar to registries for pre-analysis plans.
-Implementers	Researchers & implementers should agree to conditions that would trigger adaptation, suspension, termination.
	A TAPIR process should be documented even if the implementer proceeds without the researcher.
	Researchers should transparently report all ethics discussions even with different terms of engagement.
-Enumerators	Enumerators should undergo explicit training on sensitivity to possible enumeration-induced vulnerability.
	Researchers should balance blinding enumerators to the study's purpose with ethical considerations.
	Researchers should evaluate possible AI-induced enumeration vulnerability.
-Funders	Funders should require that vulnerability considerations are preconditions for funding.
-Society	Society should create input and accountability mechanisms for researchers.
	Society could create clearinghouses for research results to disseminate benefits of research.
	Society could also publicize perceived ethics violations with greater consistency
